# A specialized microbiome enriched in nitrification and halogen cycling potential resolved from multi-omics investigation of a Caribbean suboxic and acidic marine sinkhole

**DOI:** 10.1186/s40168-026-02564-w

**Published:** 2026-11-14

**Authors:** Meine D. Boer, Pilleriin Peets, Abraham L. van Eijnatten, Craig E. Nelson, Maria M. do Canto Brum de Menezes Pena, Szabina Karancz, Matthew P. Humphreys, Fleur C. van Duyl, Erik H. Meesters, Basten L. Snoek, Chrats Melkonian, Andreas F. Haas, Bas E. Dutilh

**Affiliations:** 1 Institute of Biodiversity, Ecology, and Evolution, Faculty of Biological Sciences, Cluster of Excellence Balance of the Microverse, Friedrich Schiller University Jena, Jena, Germany; 2 Marine Microbiology and Biogeochemistry, NIOZ Royal Netherlands Institute for Sea Research, Texel, The Netherlands; 3 Institute of Chemistry, Department of Analytical Chemistry, University of Tartu, Tartu, Estonia; 4 Theoretical Biology and Bioinformatics, Utrecht University, Utrecht, The Netherlands; 5 Daniel K. Inouye Center for Microbial Oceanography: Research and Education, Department of Oceanography and Sea Grant College Program, University of Hawaiʻi at Mānoa, Honolulu, USA; 6 Department of Ocean Systems, NIOZ Royal Netherlands Institute for Sea Research, Texel, The Netherlands; 7 Bioinformatics Group, Wageningen University and Research, Wageningen, The Netherlands

**Keywords:** Marine sinkholes, Microbiome, Metagenomics, Metabolomics, Biogeochemical cycles, Halogen cycling, Nitrification, Multi-omics

## Abstract

**Background:**

The Luymes Sinkholes, located on the Luymes Bank northwest of Saba, represent a unique marine environment characterized by distinct biogeochemical gradients. Most noticeable is an acidic (pH ≈ 6.25) and suboxic (O2 < 60 µmol/kg) submerged ‘lake’ at ~ 220 m depth exhibiting extreme deviations from standard oceanographic patterns, including elevated inorganic carbon, nitrate, and phosphate, and a lower temperature (20.3 ºC vs 26.4 ºC). To determine how the microbiome within the submerged acid lake adapted to these unique conditions, we collected 59 coupled samples of size-filtered (0.22 µm) planktonic microbes for shotgun metagenomics and solid-phase extracted dissolved organic matter for untargeted metabolomics from 21 stations at depths ranging from 2 to 600 m during a 10-day research cruise in December 2019.

**Results:**

We identified a distinct microbial community within the submerged acid lake different from the non-acidic sinkholes, with increased abundances of *Nitrospinota*, *Thermoproteota*, *Planctomycetota*, and *Actinobacteriota*. We also found three enriched classes of dissolved compounds within the submerged acid lake: (1) large, (2) low-polarity, and (3) halogenated metabolites, including iodides and halobenzoic acids. Correlations between microbial taxa and metabolite classes show potential links between nitrifying archaea and halogenated compound cycling. We further supported these findings using the functional annotation of metagenome-assembled genomes (MAGs) and protein clusters showing enrichment of nitrification and halogen cycling genes, suggesting the presence of specialized degradation and biosynthetic pathways, directly connecting microbial metabolism to the observed geochemical conditions.

**Conclusion:**

These findings demonstrate that the submerged acid lake contains specific microbially driven biogeochemical processes under acidified and low-oxygen conditions, providing a natural model for future extreme ocean acidification scenarios. With this multi‑omics approach, we were able to connect the microbial metabolism with this unique environment, thereby expanding our understanding of how both are interconnected.

Video Abstract

**Supplementary Information:**

The online version contains supplementary material available at https://doi.org/10.1186/s40168-026-02564-w.

## Introduction

Across all environments, microbes help sustain ecosystems by exchanging and transforming key elements such as nitrogen [31], carbon, and sulfur [65, 73], generating energy through chemo- or phototrophic metabolisms, and recycling nutrients. Consequently, microbes directly shape their environments by altering nutrient availability, and the composition and redox structure of the Dissolved Organic Matter (DOM) comprising the exometabolome [45, 67, 77]. In turn, microbial community composition and function are shaped by physicochemical conditions, including pH, oxygen availability, nutrient supply, and hydrodynamic connectivity [69], leading to a bidirectional relationship where each impacts the other. Comprehensive multi-omics studies, integrating metagenomics, metabolomics, and environmental measurements, are needed to resolve the intricate biogeochemical connections that shape the microbiome [31, 57, 79, 81]. Such multi-omics studies have been made possible the development of untargeted high-resolution tandem mass spectrometry of metabolomes [11] with advanced data processing tools [15, 27] and high-throughput metagenome shotgun sequencing [36, 38, 58] followed by de novo assembly and functional annotation [70]. These advances allow for increasingly detailed characterizations of potential microbe-metabolite interactions [42]. Despite these technological advances, the application of fully integrated multi-omics approaches in natural systems remains limited as the high complexity and natural variability of these systems introduce challenges that are largely absent from laboratory-based experiments [59]. Naturally stratified or physically isolated systems provide valuable opportunities to overcome some of these challenges by offering stable gradients over relatively small spatial scales [26]. Marine sinkholes can thus function as natural laboratories, as they often host sharp biogeochemical transitions.

Here, we characterize the microbial ecology of the northern sinkholes in the Luymes Bank, located at the northeast margin of the Saba Bank in the Caribbean Sea. Several of these sinkholes were previously described by Humphreys et al. [30], characterizing their complex geological origins and physical structure. Notably, Humphreys et al. [30] described an acidic (pH ≈ 6.25) and suboxic (O_2_ < 60 µmol/kg) submerged ‘lake’ starting at a depth of 220 m within the sinkhole with the deepest bottom depth (306 m). This unusual environment is thought to originate from the combined effects of an underwater CO_2_ plume and limited exchange of water with the surrounding system. The resulting acidic, low-oxygen lake is embedded within an otherwise well-oxygenated marine environment, creating a pronounced and stable biogeochemical contrast over a relatively short vertical distance. This environment offers a rare opportunity to provide insights into how microbial communities respond to acidification and reduced oxygen availability under natural conditions. As the submerged lake is both spatially compact and hydrodynamically isolated, it may serve as a natural analog for ancient ocean states characterized by enhanced CO₂ levels and widespread suboxia [60, 76], as well as for future ocean scenarios driven by ongoing anthropogenic CO₂ emissions [5, 29].

To provide these insights, we made use of a multi-omics approach. Specifically, we analyzed paired plankton metagenomic and dissolved metabolomic samples along with physicochemical parameters from multiple depths, ranging between 2 to 600 m below the ocean surface, across 21 stations. Concurrently, we used the nutrient concentrations (phosphate, nitrate, nitrite, and ammonium) and physicochemical parameters (pH, oxygen, temperature, salinity) to characterize the acid lake conditions and contrast them with other sinkholes and the overlying ocean waters. By analyzing these datasets, we could identify distinct microbial communities and metabolomic composition associated with each environment and link them to prevailing physicochemical conditions. Furthermore, because the metabolomics and metagenomics samples are spatially linked, this integrated approach lets us infer possible microbe-metabolite interactions and the biogeochemical pathways active in the acid lake, linking the observed metabolic signatures to the functional potential of the resident microbial community. This study provides new insight into how microbial communities function in naturally acidified, low-oxygen marine environments and contributes to a broader understanding of microbial roles in shaping biogeochemical cycles under changing ocean conditions. In this study, we address three central questions: (1) How does the microbial community composition differ between the acidic suboxic lake and non-acidic marine environments? (2) Which metabolic pathways are enriched in response to the extreme pH and oxygen conditions? (3) Can we link specific microbial taxa to the observed metabolomic signatures, particularly halogenated compounds? We hypothesize that nitrifying archaea and bacteria will dominate the acid lake, that acid-stress defense genes will be enriched, and that correlations between taxa and metabolite classes will reveal microbe-metabolite interactions specific to this environment.

## Methods

### Sample collection and clustering

**Fig. 1:**
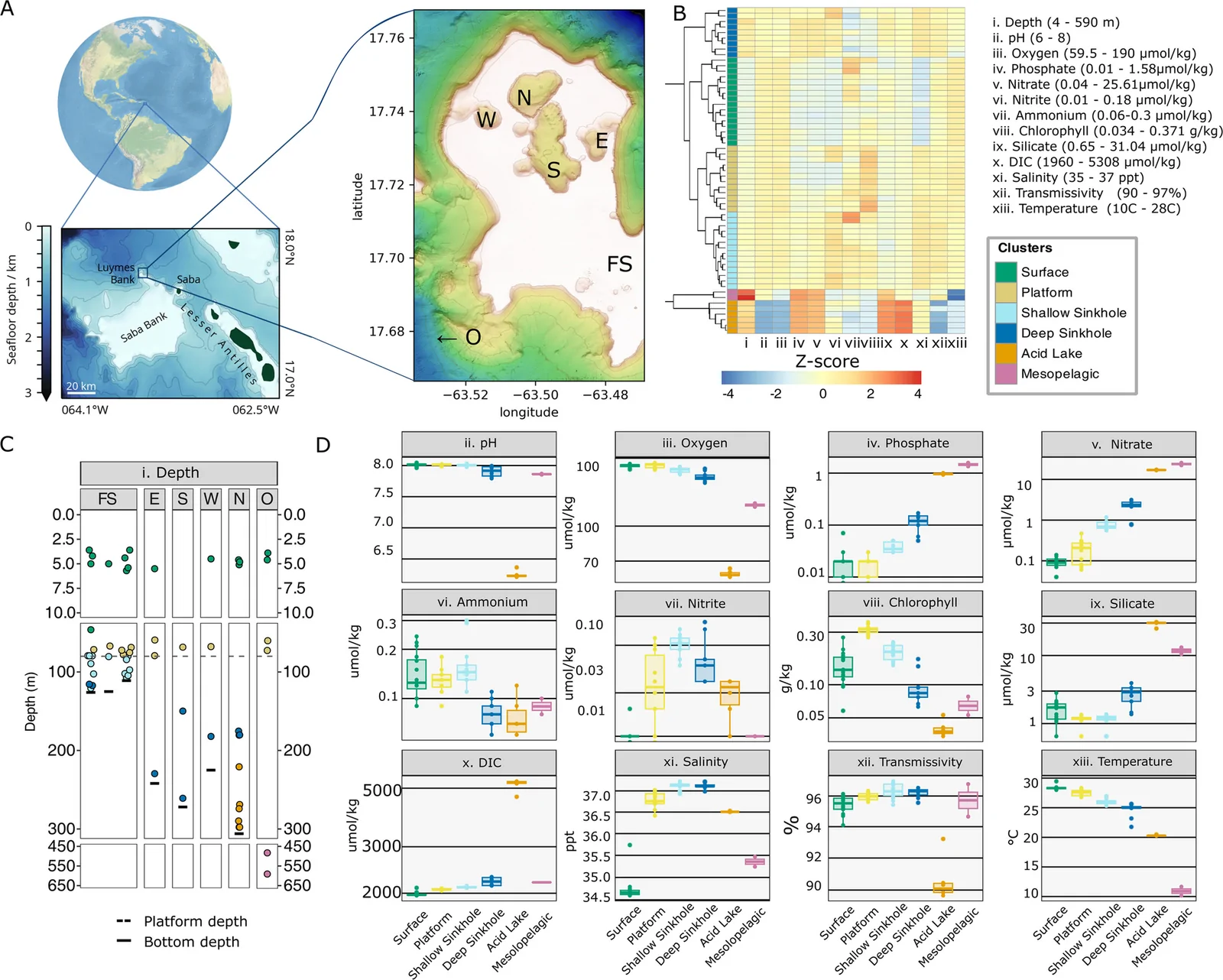
Characterization of the environment: **A** map of Luymes Bank and integrated sinkholes (modified from Humphreys et al. 2022). **B** Ward’s minimal variance clustering of physicochemical parameters; rows correspond to samples and are annotated by the six defined environmental clusters (surface (*n* = 16), platform (*n* = 12), shallow sinkhole (*n* = 14), deep sinkhole (*n* = 9), acid lake (*n* = 6), and mesopelagic (*n* = 2)). Before clustering all nutrients were log10 transformed and all factors were normalized using z-scores. **C** Sampling depths of samples grouped by sinkhole and area (FS = far south, E = east, S = south, W = west, N = north, and O = open ocean). Colored by environmental cluster, see **B**. **D** Boxplots of measured physicochemical parameters grouped and colored by environmental cluster, see legend in **B**

We collected a total of 59 paired metagenomic and metabolomic samples from 21 stations on the Luymes Bank near Saba in December 2019 (Fig. 1A). Water chemistry parameters were measured as described by [30] (Supplementary Methods, Supplementary Table S3). Based on these parameters samples were clustered using Ward’s minimal variance clustering into six environments as follows: surface (*n* = 16), platform (*n* = 12), shallow sinkhole (*n* = 14), deep sinkhole (*n* = 9), acid lake (*n* = 6), and mesopelagic (*n* = 2). More detailed information on sample collection and storage can be found in the Supplementary methods.

### Microbial abundance measurements

Flow cytometric enumeration of prokaryotes (including bacteria and archaea) and viruses was performed at the NIOZ according to the protocol by Brussaard et al. [6] and using a TE buffer at pH 8.2. Samples were diluted in TE buffer and stained with the nucleic acid-specific green fluorescence dye SYBRGreen-I (10–5 dilution of commercial stock, Sigma-Aldrich, USA) for 15 min at room temperature for prokaryotes and for 10 min at 80 °C for viruses. Samples were then analyzed using a FACSCalibur flow cytometer with a 488 nm laser (BD Biosciences, USA).

### DNA isolation and sequencing

Total DNA was extracted from seawater using the NucleoSpin Tissue DNA extraction kit following manufacturer instructions with some small adjustments (see supplementary methods section: “DNA isolation”). The isolated DNA was measured using the Denovix DS-11, and ranged from 1.7 to 12 ng/µL. Genomic DNA libraries were subsequently prepared using the TruSeq DNA Nano LT Sample Preparation Kit (Illumina), whereby DNA was mechanically fragmented, end-repaired and 3′-adenylated, ligated to indexed adapters, size-selected by bead-based purification, PCR-amplified, and subsequently sequenced on the Illumina NovaSeq 6000 sequencing platform with a mean read length of 145.2 bp a mean of 36 M reads per sample (Supplementary Fig. S1). For further details on DNA extraction, library preparation and sequencing please see Supplementary methods.

### Metagenome assembly, classification, and annotation

The reads were filtered, assembled, and binned using the MetaCascabel pipeline (v3.0, [1]. The reads were trimmed and filtered using Trimmomatic (v0.39), trimming the Illumina adapter and poor quality ends (Phred Q-score < 3) and removing reads with a stretch of Phred Q-score < 22 for 5 bp or length < 100 bp. Quality was assessed using FastQC (v0.11.9, [3], Supplementary Table S1). After quality assessment, the reads were assembled with MetaSPAdes (v3.15.5, [48] using the default parameters and assessed contiguity, unknown bases, and GC content using QUAST (v5.2.0, [24]. After assembly, the contigs were binned into metagenome-assembled genomes (MAGs) using Binsanity (v0.5.4, [21], CONCOCT (v1.1.0, [2], MaxBin (v2.2.7, [78], and MetaBAT 2 (v2.12.1, [34] and their output merged and filtered using DAS Tool based on completeness, bin size, and redundancy (v1.1.5, [66]. We also assessed the quality of the MAGs (completeness, contamination, strain heterogeneity) using CheckM (v1.2.1, [53], Supplementary Fig. S2).

The MAGs from the separate assemblies were then dereplicated using dRep (v3.4.5, [50], Supplementary Table S2) and the relative abundances of the MAGs within samples were calculated through read mapping with bwa-mem2 (v2.2.1, [74]. The taxonomic profiles of the metagenomes were determined by classifying and quantifying MAGs, contigs, and reads using the Read Annotation Tool RAT (v6.1, [25], which automatically integrates different assembly levels, and the Genome Taxonomy Database [52], version R202. Alpha and beta diversity were calculated using the phyloseq package (v1.41.1, [44] in R. Statistical differences between environments were tested using TukeyHSD based on the 59 samples over the six environments (Fig. 1). Functional annotation of the MAGs was done using the Functional Annotation pipeline of NIOZ [1] genes were predicted using Prodigal, followed by alignment using DIAMOND and HMMER [7, 17] and annotation to the KEGG Orthology [33], PFam [54], arCOG [41], and COG [19]databases. Additionally, we used the contig annotations from Prodigal, which was run as part of the taxonomic classification pipeline (see above). Subsequently, proteins were clustered using MMseqs2 (v15.6, [68]) and annotated with eggNOG-mapper (v2.2, [9]).

### Untargeted metabolomics

Measurements were performed on the ultra-high performance liquid chromatography system (UHPLC): Agilent 1290 Infinity II equipped with a thermostatted (30 °C) auto-injector and column compartment, coupled to a Q-Exactive Plus Orbitrap Mass Spectrometer (Thermo Fisher Scientific, Bremen, Germany) equipped with an Ion-max source with a HESI (Heated Electrospray Ionization) probe. Chromatographic separation was achieved with a C18 core–shell column (Kinetex, 150 × 2 mm, 1.8 µm particle size, 100 Å pore size, Phenomenex, Torrance, USA) and 17-min gradient (A: H_2_O + 0.1% formic acid and B: Acetonitrile + 0.1% FA at a flow rate of 0.5 mL/min). Metabolites were eluted from the samples using the following program: 0.5 min of 5% B, followed by a linear gradient to 50% B over 7.5 min, further followed by a 2 min gradient to 99% B. This step is followed by a wash phase of 7 min at 99% B and a re-equilibration at starting conditions for 8 min. LC–MS data were acquired in positive ionization mode using a data-dependent approach for fragmentation spectra of the top 5 highest intensity peaks. Instrument-specific spectrum data were converted into mzXML format using msConvert and MS1 spectra were consensus aligned across samples using MZmine (version 4.2.3, [64]). LC–MS ion features were annotated and compound class vectors predicted using CSI:FingerID [15, 28]and CANOPUS [14] inside SIRIUS (version 5.8.3, [15]). To define a sample-level chemical probability profile using CANOPUS we applied the chemical characteristics vector (CCV) approach adapted from previous work [55]. Briefly, CCV averages compound class probabilities assigned to each ion feature by CANOPUS across all ion features in a sample to derive a bulk compound class probability profile for each sample. Here we improved on the original method by using weighted averaging to incorporate normalized LC–MS peak areas (representing relative compound abundances within each sample by dividing each ion peak area by the total sample peak area) into the averaging process. Statistical analysis was conducted in R (version 4.4.2.).

## Results

### The submerged acidic lake constitutes a unique marine environment

The Luymes Banks consist of a carbonate platform raised from the ocean floor, up to a depth of 60–80 m below the ocean surface and features multiple sinkholes with depths of up to 475 m below the ocean surface across their 66 km^2^ area [71]. We performed a characterization of seven of these sinkholes and adjacent open-ocean stations using multivariate clustering of hydrographic and biogeochemical parameters (phosphate, nitrate, nitrite, pH, depth, dissolved oxygen, transmissivity, salinity, and chlorophyll,(Fig. 1B, C). We excluded total alkalinity from the clustering analysis due to its strong collinearity with dissolved inorganic carbon (DIC, Supplementary Fig. S3). The multivariate clustering revealed six distinct environments: surface, platform, shallow sinkhole, deep sinkhole, acid lake, and mesopelagic (Fig. 1 C, D). Each cluster delineates a discrete physicochemical biome characterized by a unique combination of water column properties (Supplementary Fig. S3B), that approximately correspond to increasing depths (surface: *n* = 16, mean = 2.7 m, std = 3 m; platform: *n* = 12, mean = 70 m, std = 6 m, shallow sinkhole: *n* = 14, mean = 92 m, std = 10 m; deep sinkhole: *n* = 9, mean = 177 m, std = 47 m; acid lake: *n* = 6, mean = 275 m, std = 30 m; and mesopelagic: *n* = 2, mean = 536 m, std = 76 m). With the notable exception of the acid lake, the six different environments correspond to regular oceanographic patterns including higher nutrient levels in deeper, cooler water, and a chlorophyll max at intermediate depth [12, 40]. Samples collected near the surface (surface) were similar across all sinkholes and the open ocean, featuring high light, temperature, and dissolved oxygen with low concentrations of salinity, inorganic nutrients, and chlorophyll (Fig. 1C, D, E). Samples collected just above the platform depth at a depth of 60–80 m (platform), had higher salinity (mean = 37 ppt,std = 0.18 ppt) than the surface samples (mean = 34.7 ppt; std = 0.27 ppt), the highest chlorophyll concentrations of all environments (mean = 0.31 µg/kg, std = 0.03 µg/kg), and marginal increases in oxygen (+ 0.3 µmol/kg), nitrate (+ 0.11 µmol/kg), and nitrite (+ 0.035 µmol/kg) relative to the surface. The conditions within the sinkholes (shallow sinkhole, deep sinkhole, and acid lake) diverged from platform waters with increased salinity, nitrate, and phosphate. These patterns were consistent across both northern and southern sinkholes. The most striking deviations from the general pattern were observed in the acid lake environment, located within the northern sinkhole (latitude = 17.7464º, longitude = − 63.4985º). Starting at 220 m depth, the submerged acid lake showed a nearly two-unit pH decline (8.02 to 6.25), two-fold increase in inorganic carbon (2083 to 5164 µmol/kg), lower temperature (26.4 ºC vs 20.3 ºC), low oxygen (177 vs 60 µmol/kg), and an order-of-magnitude increase in nitrate (1.55 vs 17.0 µmol/kg) and phosphate (0.96 vs 9.6 µmol/kg) relative to similar depths in the other sinkhole environments (Fig. 1B, D). As these unique conditions are virtually non-existent in other marine systems, the acid lake is of particular interest for further analysis. Following known oceanographic patterns [57, 61], the mesopelagic waters (500–600 m) were very distinct from all others samples, with temperatures near 10 °C, phosphate and nitrate concentrations 10-fold higher, and the second-lowest oxygen concentrations after the acid lake.

### A distinct microbiome in the acid lake environment

To understand how abiotic factors shape microbial communities, we compared the taxonomic composition of all six defined environments. Overall, cell numbers decreased with depth (Supplementary Fig. S4C), consistent with previous findings that surface waters tend to support higher microbial density [18]. Genus richness (Chao1), calculated using phyloseq (v1.41.1, [44]) in R, showed a slight decrease within the acid lake environment compared to the other sinkhole environments (shallow sinkhole and deep sinkhole, Supplementary Fig. S4A). The acid lake also exhibited lower genus evenness (Simpson) than the other environments (Supplementary Fig. S4B), suggesting that, despite comparable diversity, the relative abundances of taxa are more skewed.

**Fig. 2:**
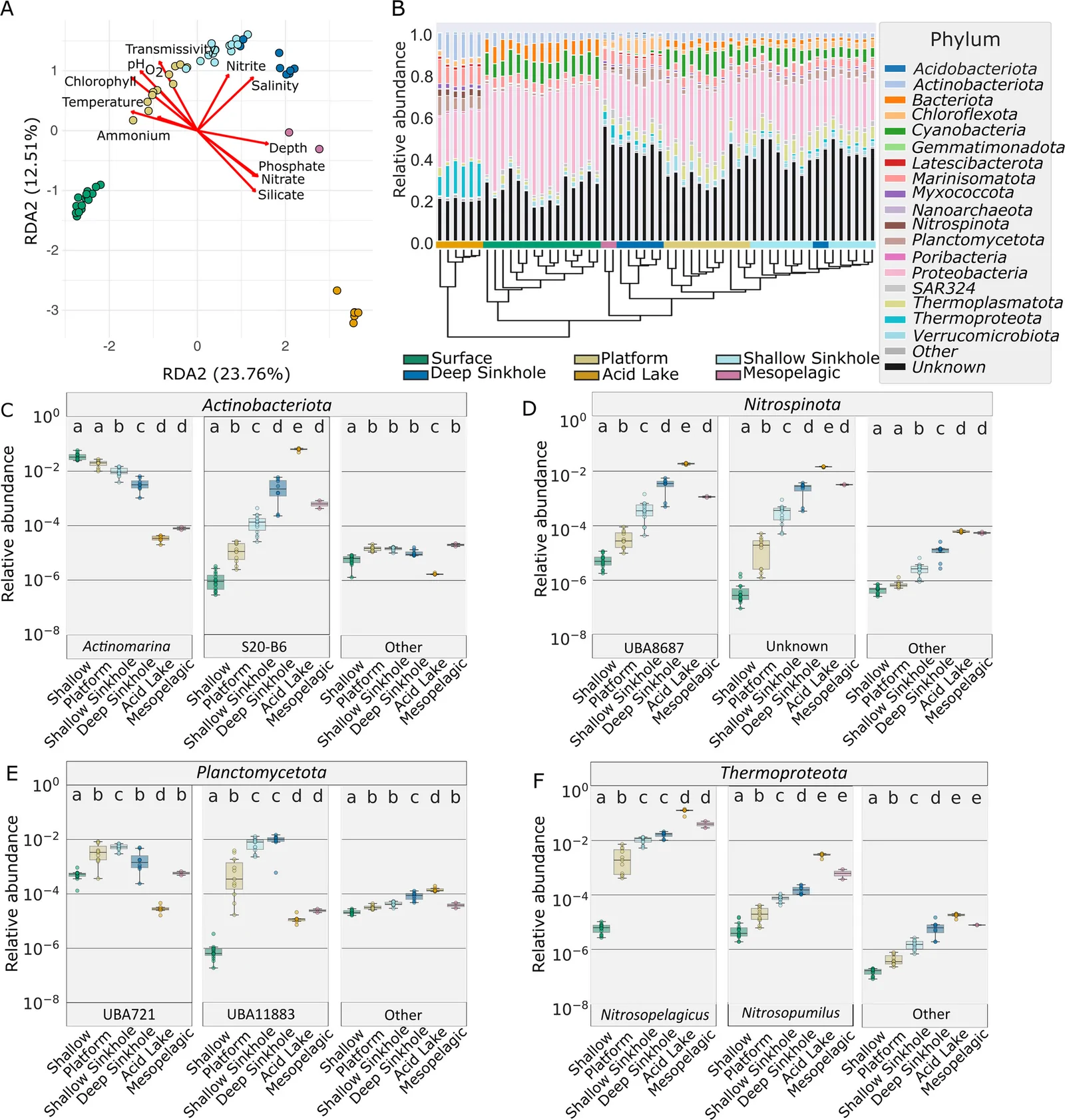
Characterization of microbiomes from 59 metagenomics samples: **A** ordination of 59 microbiomes colored by environments defined by physicochemical clustering (Fig. 1): surface (*n* = 16), platform (*n* = 12), shallow sinkhole (*n* = 14), deep sinkhole (*n* = 9), acid lake (*n* = 6), and mesopelagic (*n* = 2). Red arrows show the direction and strength of association between environmental measurements (pH, oxygen, temperature, depth) and microbial community patterns calculated using Redundancy Analysis (RDA) from the vegan package in R [49]. **B** Relative abundance of the 20 phyla with highest mean relative abundance across all samples clustered by microbiome profile and colored by environment. All other phyla are aggregated as “Other”. **C**–**F** Relative abundance of the three genera with highest relative abundance in each phylum: **C** *Actinobacteriota*, **D** *Nitrospinota*, **E** *Planctomycetota*, and **F** *Thermoproteota* grouped based on environment. Letters above the boxplots show significantly different environments (*p* < 0.05, TukeyHSD) determined using the stats package in R

To visualize variation in taxonomic structure of microbial communities among all 59 samples we used redundancy analysis (RDA; Fig. 2A). This revealed that beta diversity differed between environments, with a slight overlap between the shallow and deep sinkhole environments and a strong separation of the acid lake. Through RDA analysis we found that pH, oxygen, temperature, and depth were significant (ANOVA permutation test, *p* < 0.001, *n* = 59), in line with those factors being the most illustrative of the unique conditions of the acid lake environment.

This relation between environment and microbial community composition was further revealed when we considered the taxonomic composition of the samples (Fig. 2B). Several samples also showed relatively high fractions of unknowns that could not be annotated even at domain level, suggesting their origin might be viral or eukaryotic, though attempts at annotation remained unsuccessful. The acid lake samples form a distinct cluster characterized by low pH, suboxic conditions, and elevated nutrients. Specifically, the samples from the acid lake contained significantly more *Actinobacteriota* (Fig. 2 C, 2.5–6x, *p* < 10^–5^, TukeyHSD), *Nitrospinota* (Fig. 2D, 5–5,000 × *p* < 10^–5^), *Planctomycetota* (Fig. 2E, 2–7x, *p* < 10^–5^), and *Thermoproteota* (Fig. 2 F, 3–3000x, *p* < 10^–5^) than other samples, and significantly fewer *Cyanobacteria* (Supplementary Fig. S5A, 0.05–0.008x, *p* < 10^–5^) and *Thermoplasmatota* (Supplementary Fig. S5B, 0.3–0.6x, *p* < 10^–5^). The increased presence of *Actinobacteriota* is primarily due to the genus S20-B6, which is a member of the order *Acidimicrobiales*. This genus is prevalent in the acid lake, whereas the genus *Actinomarina* is more common deeper in the non-acidic deep sinkholes (deep sinkhole, 2 C). Notably, S20-B6 was previously detected at > 200 m depth and was linked to microbial nitrate reduction, though literature shows intergenus variation [62] and functional annotation did not identify the associated *nar* gene (see “Functional annotation shows increased nitrification, halogen recycling, and acid resistance” section). Additionally, bacteria of this genus have shown high versatility in the utilization of sugars [62], which may provide a competitive advantage within the acid lake. In the phylum *Nitrospinota*, the dominant genus in the acid lake environment is UBA8687 (Fig. 2D), an uncultured member of the *Nitrospinaceae* family. Other *Nitrospinota* genera were also more abundant (5–2860x, *p* < 10^–5^, TukeyHSD) in the acid lake environment than in the other environments. *Nitrospinota* is a well-known phylum of nitrite-oxidizing bacteria that plays an important role in the conversion of nitrite to nitrate [4, 13, 37]. The most abundant genus within the acid lake environment was *Nitrosopelagicus* from the phylum *Thermoproteota* (Fig. 2F). This genus contains several ammonia-oxidizing archaea typically found at depths of 200 m or less [56, 63]. However, their abundance in the deeper acid lake (220–297 m) might be explained by their known fitness in low-pH (6.5–6.2) and high-nitrate (16.0–17.5 μmol/kg) environments [56].

The high abundances of *Thermoproteota* and *Nitrospinota* suggest that ammonia oxidation by *Nitrosopelagicus* and subsequent nitrite oxidation by *Nitrospinota* members may contribute to the increased nitrate levels in the acid lake (Fig. 1B, D). Since both nitrite and ammonia oxidation consume oxygen, and ammonia oxidation promotes acidification [46, 82], these microbial processes would reinforce the unique conditions found within the acid lake.

### Co-occurrence analysis reveals distinct communities

**Fig. 3:**
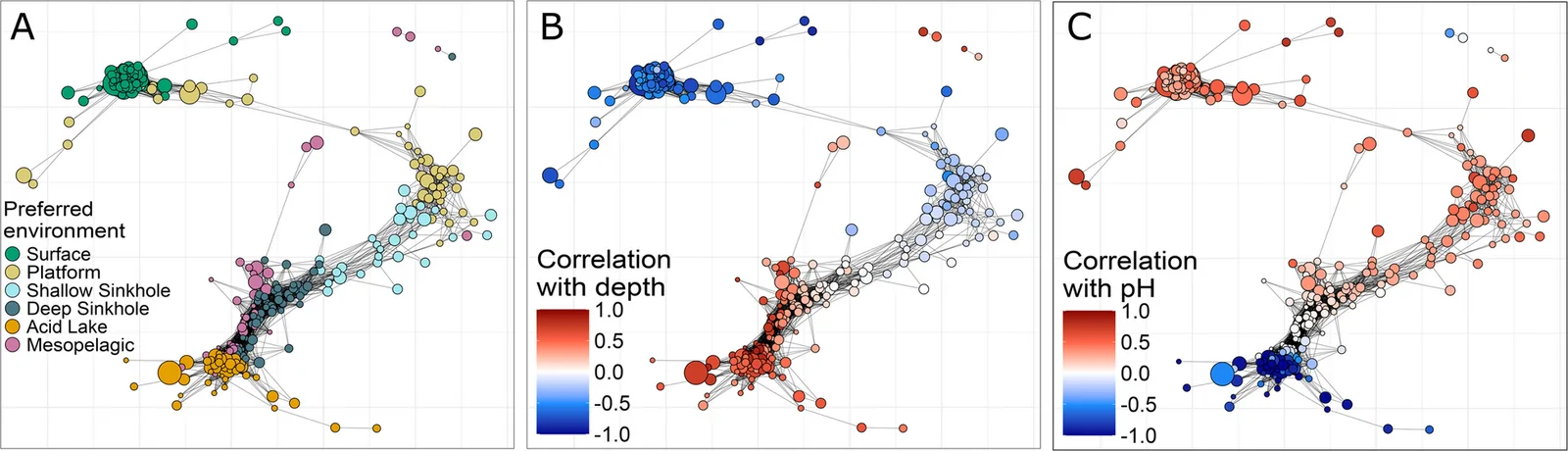
SpeSpeNet correlation networks: **A–C** correlation networks of 316 genera colored by **A** environment with highest relative abundance, **B** Pearson correlation with depth, and **C** Pearson correlation with pH. Networks were generated using SpeSpeNet [72]. Edges show co-occurrence based on Spearman rank correlation (ρ > 0.5) with nodes filtered for abundance (> 0.001 in > 1 samples), prevalence and covariation (> 0 in > 4 samples), and covariation (> 1 correlation with ρ > 0.5). The overall layout is generated using a Fruchterman-Reingold algorithm

Next, we tried to understand community structure and response to environmental factors from the perspective of the microbial genera to complement our sample-level approaches described in the previous section. We investigated how the environment drives microbial co-occurrence within the different environments we defined within the Luymes ecosystem. We created a co-occurrence network of the abundances of all genera across the 59 metagenome samples using SpeSpeNet [72], Fig. 3). This network connects the genera represented by nodes that are sized according to their abundance. Distances between nodes are determined by a matrix of Spearman correlation coefficients, with edges visualized when rho > 0.5. This yielded a network with 316 nodes (genera) and 7074 edges (co-occurrences). By assigning different colors to the genera in the network based on the environment in which their relative abundance was highest, we observed a clear separation of the surface-associated genera while genera preferring other environments showed a more contiguous structure but still formed distinct communities (Fig. 3A). Correlating the relative abundance of the genera with depth (Fig. 3B) and pH (Fig. 3C) shows how the non-surface associated genera are on a gradient corresponding to depth, while the pH drives microbial co-occurrence in the deepest environments (deep sinkhole, acid lake, mesopelagic). Correlation of the genera with oxygen and transmissivity showed the same pattern as pH (Supplementary Fig. S6GM), likely due to interdependency of these factors (Supplementary Fig. S3). Correlation with phosphate, total alkalinity, silicate, and to a lesser extent nitrite and ammonium showed an inverse pattern (Supplementary Fig. S6FH). Correlation with pressure, chlorophyll, and temperature showed patterns comparable to depth (Supplementary Fig. S6CL). Overall, these results demonstrate a strong separation between the surface-associated genera and the other genera within the Luymes ecosystem, which is organized following the depth gradient with an additional effect of the low pH and oxygen conditions from the acid lake environment.

### The acid lake metabolome is enriched with high-mass, low-polarity, and iodide compounds

**Fig. 4:**
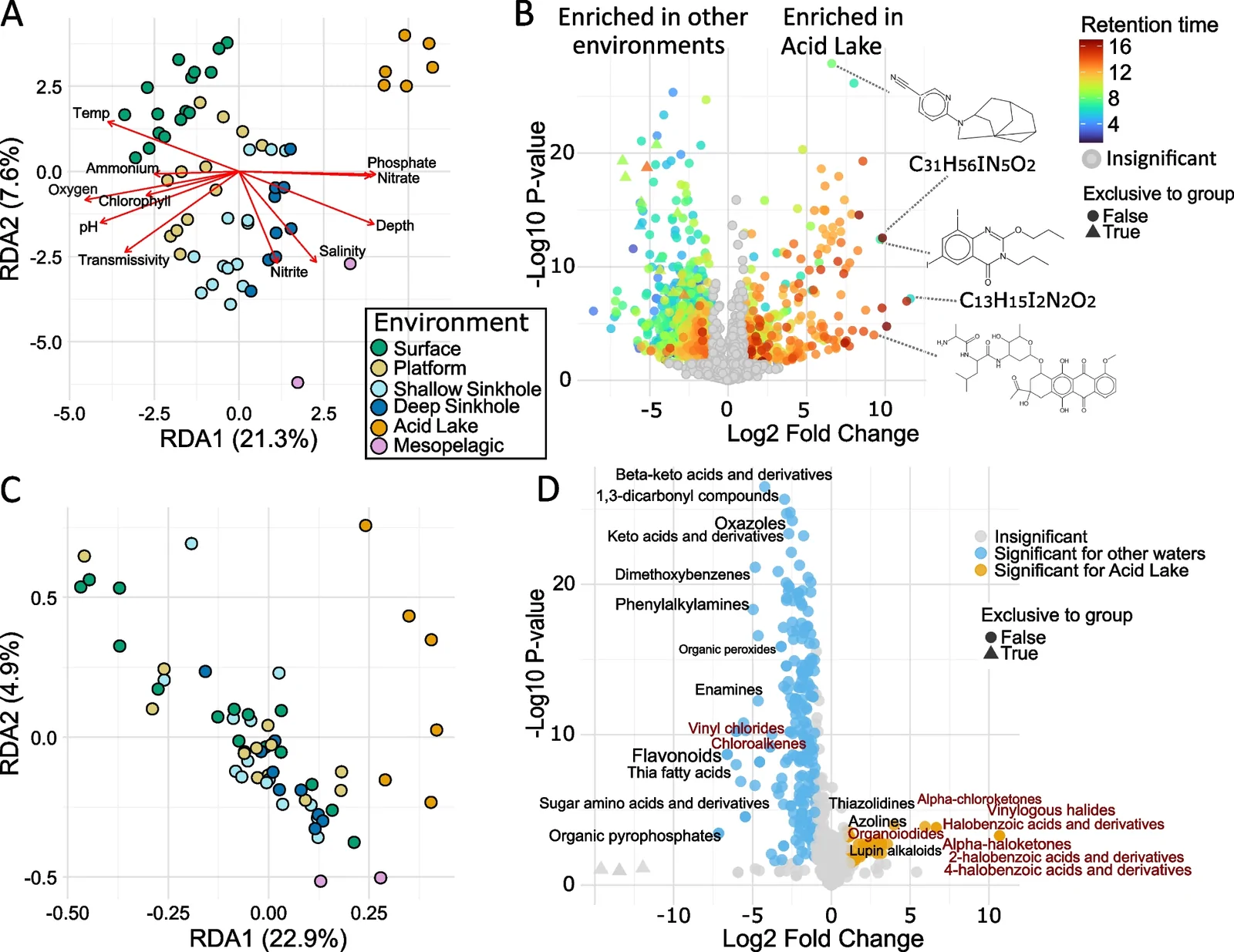
Untargeted metabolomics with LC-HRMS: **A** ordination of log_10_-transformed peak areas of the 2746 ion features with corresponding MS2 data within the 59 metabolomics samples, colored by environments defined by physicochemical clustering (Fig. 1). Arrows show the effects of the explanatory variables within the linear space using Redundancy Analysis (RDA) generated by the vegan package in R. **B** Volcano plot of chromatographic peak areas. The *x*-axis shows a log2-fold change of log_10_-transformed mean peak area of acid lake versus other environments. The *y*-axis shows *p*-values calculated with unpaired *t*-test on log-transformed data to correct for non-normality and using Benjamini–Hochberg to correct for false discovery rate using the stats package in R. Coloring shows retention time as a proxy for metabolite polarity for ion features where the mean peak area was 2 × higher in one or the other habitat. For the five compounds that were highly enriched in the acid lake, the chemical structure (when available) or formula is shown. **C** Ordination of intensity-weighted CCV chemical class profiles predicted from MS2 data within the 59 metabolomics samples, colored by the sampled environment. **D** Volcano plot using CCV chemical class profiles to detect annotated compound classes enriched in acid lake. Gray color for the points indicates insignificance (*p* > 0.05, unpaired *t*-test) or quantitative similarity (0.5 < difference < 2x) when comparing acid lake vs other environments, orange shows compound classes enriched (> 2 × difference and *p* < 0.05) in acid lake, and blue classes enriched (< 0.5 × difference and *p* < 0.05) in other waters. Labels for distinct halogenated compounds are colored red; the rest of the compound classes are marked in black. A table of all compound classes is provided in Supplementary Table S5

To determine the chemical composition of the different environments, we characterized their exometabolomes using LC–MS/MS-based untargeted metabolomics of solid-phase extracted DOM. While bulk water chemistry establishes the physicochemical constraints of each environment (pH, oxygen, nutrients), the exometabolome captures the dynamic organic component that directly mediates microbe-microbe and microbe-environment interactions. Similar to the microbial compositions, distinctive exometabolome profiles were observed, primarily separated by pH, O_2_, and depth (Fig. 4A). The acid lake was enriched for compounds with high retention times, indicative of lower polarity in reverse-phase chromatography [20] and depleted for compounds for low retention times (Fig. 4B, Supplementary Fig. S7C). Compounds enriched in the acid lake also showed greater molecular masses than those from other environments (Supplementary Fig. S7AB). High-mass apolar compounds are generally more resistant to microbial degradation [43], suggesting that reduced mixing combined with microbial specialization may allow the acid lake to function as a sink for recalcitrant DOM. Conversely, the low abundance of small low-polarity compounds may reflect enhanced degradation and remineralization of smaller, high-polarity compounds [22]. We identified 67 out of 327 (20.4%) enriched compounds (log2 fold change > 1, *p* < 0.05, unpaired *t*-test) in the acid lake as iodides, compared to only 2 out of 1050 (0.19%) in other waters (rank1 formula from SIRIUS). Other halogenated classes did not show comparable enrichment, with only two chlorides (0.61%) and 19 fluorides (5.8%) enriched in the acid lake compared to 12 chlorides (1.1%) and 85 fluorides (8.1%) in the other environments. This selective enrichment is notable because iodide ions occur at much lower natural concentrations than chloride or bromide [39], making marine organic iodides relatively rare and underrepresented in annotation databases [23, 39]. Consequently these compounds are often difficult to detect and annotate. Their enrichment in the acid lake therefore provides an opportunity to expand our knowledge of this underrepresented compound class. While compound size and polarity provide useful chemical descriptors, biological function primarily depends on molecular structure. Using the CSI:FingerID structural prediction tool within SIRIUS we predicted probable structures for 2470/2746 in all environments, and 253/327 enriched in the acid lake, although only 18 had COSMIC confidence score > 0.5 [28]. To further characterize chemical composition despite limited structural annotation, we applied intensity-weighted chemical characteristics vectors (CCV) derived from SIRIUS + CANOPUS predictions [55]. This method assigns compounds to their probable chemical ontology class, allowing compounds without full structural annotation to contribute to the chemical profiling of samples. When we compared samples based on this compound class chemical space (Fig. 4C), non-acid lake environments separated less strongly than when we considered individual features (Fig. 4A). However, acid lake samples remained clearly distinct, indicating that the observed shift reflects broader chemical characteristics rather than only differences in feature intensity.

We found a reduction in beta-keto acids, dimethoxybenzenes, and phenylalkylamines within the acid lake (Fig. 4D). Conversely, enrichment was observed for multiple halogen-related compound classes (Fig. 4D). To select the compound classes most relevant for separating the acid lake from other waters, we used recursive feature elimination (RFE). Based on tenfold cross-validation, 200 classes were flagged as important, meaning they appeared at least once within the top 100 ranked features, while from this set 23 were enriched in acid lake (CCV value at least 2 × higher in acid lake). Of the 23 selected compound classes that were enriched in the acid lake, 10 were halogen-related (Supplementary Table S4). Notably, several classes were represented by a single compound, such as vinylogous halides, 2-halobenzoic acids, and alpha-haloketones. Broader compound classes displayed a greater number of individual LC–MS ion features, such as halogenated fatty acids (11 features), organoiodides (69 features), and alkyl iodides (22 features; Supplementary Table S4, Supplementary Fig. S8A). Among the alkyl iodide features, 8 out of 22 demonstrated significant enrichment in acid lake samples (Supplementary Table S4, Supplementary Fig. S8C).

We could not annotate all ion features classified as halogenated compounds with a final structure. In some cases, annotation was hindered by limited fragmentation data, e.g., the compound with the formula C_13_H_15_I_2_N_2_O_2_ (Fig. 4B). In other cases, e.g., the compound with the best matching chemical formula C_31_H_56_IN_5_O_2_, the LC–MS feature exhibited extensive fragmentation, but structural annotation was still not possible. Interestingly, 74 features out of 327 (22.6%) enriched in the acid lake did not end up with any structural annotation, while the number of unannotated features for the other group was only 44 out of 1050 (4.2%) features. This suggests that the acid lake contains more novel compounds that cannot be found in any of the databases than the other environments. While further annotation and confirmation of these compounds was beyond the scope of this study, we can still gain insight through their connection with the microbial profile and their functional potential to further explore their origins.

### Metabolomics correlated with microbial taxonomy

**Fig. 5:**
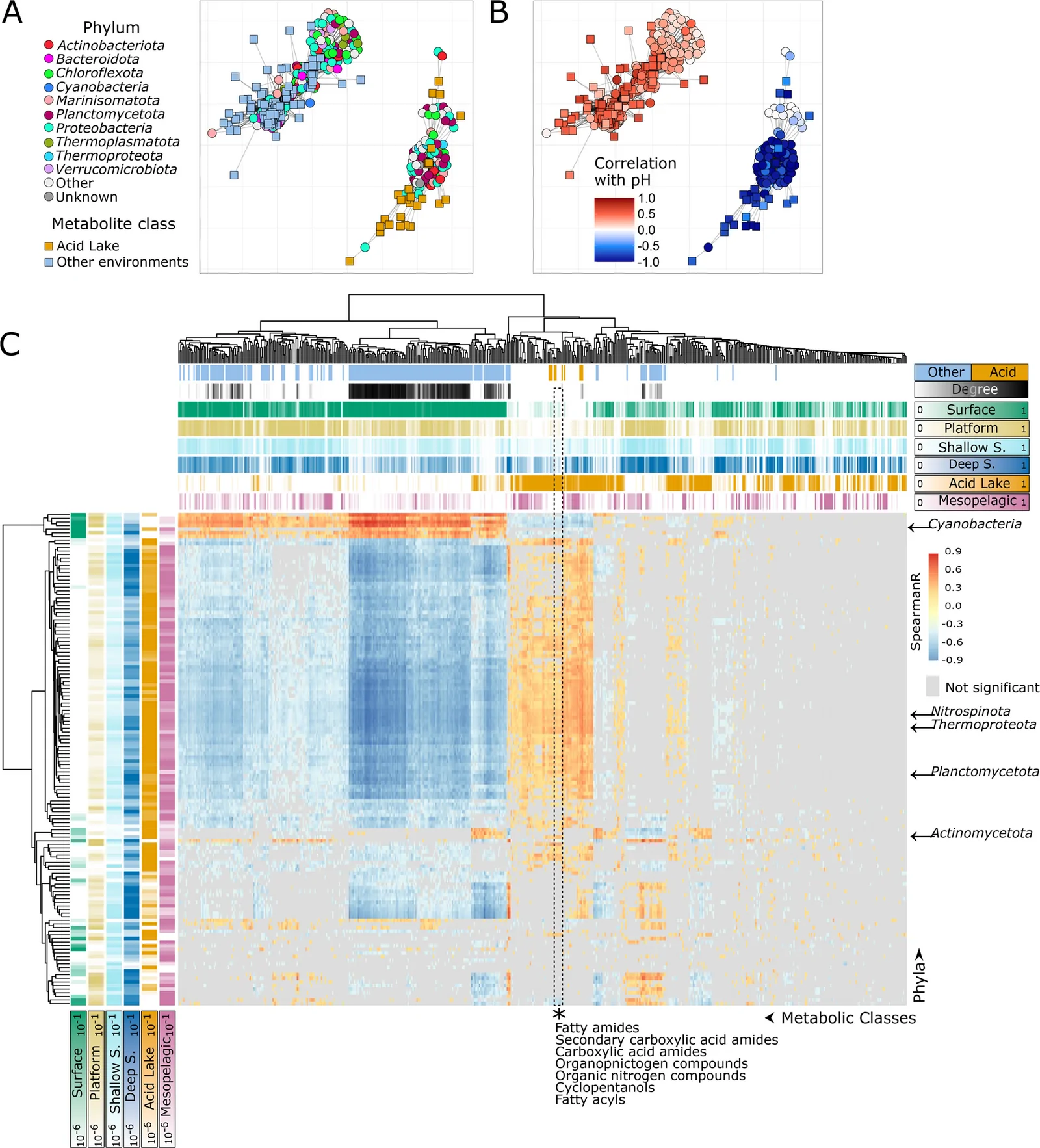
Metabolome-metagenome correlation: taxonomic-metabolomic correlation network colored by **A** metabolite class or phylum or **B** correlation with pH. The microbe-metabolite correlation network was made by adapting code from SpeSpeNet [72]. Network properties were calculated using the igraph package (Antonov et al., 2023). Edges show co-occurrence based on Spearman rank correlation (ρ > 0.5) with nodes filtered for abundance (> 0.001 in > 1 samples), prevalence and covariation (> 0 in > 4 samples), and covariation (> 1 correlation with ρ > 0.5). **C** Correlation heatmap of the Spearman correlation between phyla annotated with normalized abundance in the six environments and for metabolic classes present in SpeSpeNet their network and degree (number of connections). Correlations between phyla and CANOPUS compound classes for correlation heatmap were calculated using SciPy [75]

To link chemical composition with microbial community structure, we assessed associations between microbial taxa at the genus level and metabolite classes using a Spearman’s rank correlation-based network (ρ > 0.5). This revealed two taxonomic-metabolomic sub-networks (Fig. 5A). The first sub-network consists of 86 taxa, 29 metabolite classes, and 471 taxa-metabolite interactions, and is negatively correlated with pH (Fig. 5B). The elements in this sub-network are found predominantly in the acid lake. The second sub-network contains 228 taxa, 153 metabolites, and 12,453 metabolite-taxon edges, is positively correlated with pH, and the taxa and metabolite classes from this network are predominantly found in the non-acid lake environments.

We also created a hierarchically clustered heatmap of all correlations between phyla and metabolic classes (Fig. 5C). This revealed several distinct blocks of positively and negatively correlating taxa-metabolite class connections, which are related to their relative abundances in different environments. Several of the acid lake-associated taxa (*Thermoproteota*, *Nitrospinota*, *Actinobacteriota*, *Planctomycetota*) can be found separated from non-acid lake phyla, and showing high similarity to other phyla abundant in the acid lake. The metabolite classes of the two networks can be identified within this heatmap. A clear separation of acid lake vs. non-acid lake is visible, with the strongest correlations being associated with higher degree within the network. Overall, the networks and heatmap analyses suggest that the unique conditions within the acid lake are a major driver of the correlations suggesting a distinct system of taxa-metabolite class interactions.

### Functional annotation shows increased nitrification, halogen recycling, and acid resistance

Next, we linked environmental chemistry to microbial functional potential using two complementary approaches: (1) functional annotation of metagenome-assembled genomes (MAGs) and 2) functional annotation of all contigs via protein clustering to optimally utilize the strong taxonomic signal of MAGs and the increased coverage of contigs. For the MAG-centered approach, we selected 102 dereplicated MAGs based on completeness and contamination (Supplementary Fig. S2). These MAGs were 89 ± 7.14% complete, showed 3 ± 4.6% contamination, and had an average size of 2.7 ± 1.4 Mb (Supplementary Table S2). For the contig-based approach, predicted protein sequences were clustered into over 4 million protein clusters of which 2 million could be annotated. Gene abundances were estimated from length-normalized contig abundances to generate environment-specific functional profiles (Supplementary Fig. S10).

**Fig. 6:**
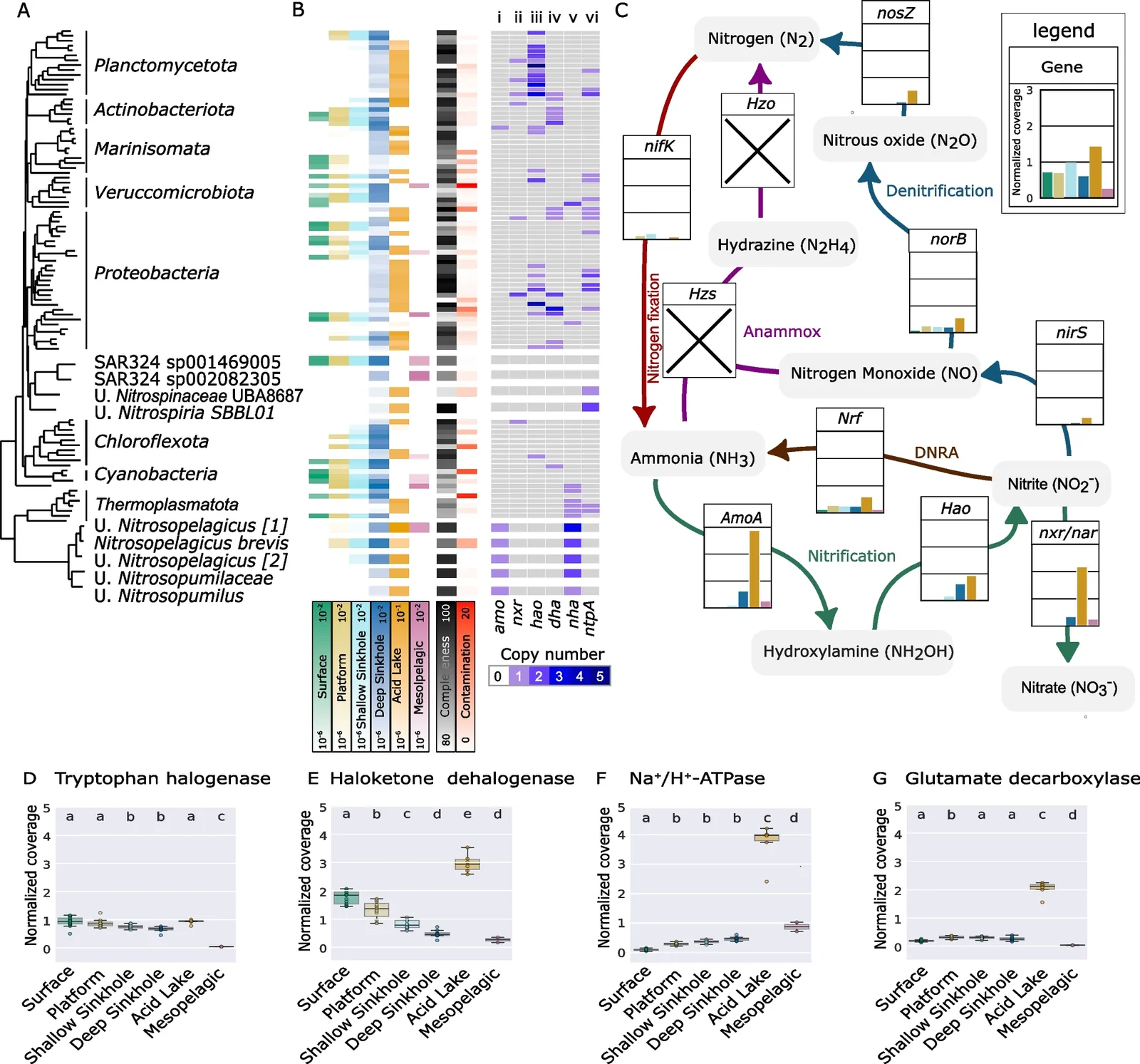
Functional annotation of metagenomes: **A** phylogenetic tree of 102 dereplicated metagenome assembled genomes (MAGs) based on concatenated alignment of 396 marker genes using Phylophlan (Asnicar et al., 2020) with default parameters. A fully annotated tree can be seen in Supplementary Figure S9. **B** Heatmap showing log-scaled relative abundances colored by the six different sampled environments, followed by completeness in black and contamination in red, and the copy numbers of marker genes related to nitrification (i–iii), halogen metabolism (iv), and acid stress defense (v–vi). C–G) Normalized coverage of **C** nitrogen cycle marker genes, **D** Tryptophan halogenase, **E** Haloketone dehalogenase (*dha*), **F** Na^+^/H^+^-ATPase (*nha*), and **G** Na^+^/H^+^ antiporter (*ntpA*). Normalized coverage is calculated based on contig abundance from RAT normalized by contig length, summed by sample, and averaged over the environment. Letters show significantly different environments (*p* < 0.05, TukeyHSD)

We first examined nitrogen cycling because two phyla enriched in the acid lake, *Thermoproteota* and *Nitrospinota*, are key nitrifying phyla. Two very abundant *Thermoproteota* MAGs in the acid lake: U. *Nitrosopelagicus* [1] (20%) and U. *Nitrosopelagicus* [2] (6%, Supplementary Fig. S11) encoded the ammonia oxidation gene *amo* (Fig. 6B), consistent with the high *amo* abundance in protein clusters (Fig. 6C). Dominant *Nitrospinota* MAGs (SBBLO1 0.2% and UBA8687 0.3%) lacked canonical *nxr* genes required for nitrite oxidation (Fig. 6B, [4], something previously observed [35, 80]. Analysis of protein clusters revealed additional *nxr* genes with 10-fold higher abundance in the acid lake than in other environments (*p* < 10^–5^, Tukey HSD, Supplementary Fig. S12) suggesting wider nitrite oxidation distribution amongst taxa.

The gene encoding the intermediate step (*hao*) was detected in both MAGs and protein clusters (Fig. 6B, C). The high abundances of *amo*, *hao*, and *nxr* suggest that the acid lake community is capable of full nitrification despite extreme acidity. Denitrification genes (*nirS*, *norB*, *nosZ*) were slightly more abundant within the acid lake, whereas nitrogen fixation and anaerobic ammonia oxidation genes were absent (Fig. 6C). We also found that the S20-B6 MAG (completeness = 97.44%) did not contain the *nar* gene, suggesting that it may not be capable of nitrate oxidation, unlike other S20-B6 species [62].

We next examined carbon fixation pathways in our most abundant MAGs (Supplementary Fig. S9B). *Nitrosopelagicus* (*Thermoproteota*), the dominant ammonia-oxidizing archaeon in the acid lake, lacks Rubisco but does encode incomplete autotrophic pathways. In contrast, several *Acidimicrobiales* (*Actinobacteriota*) and *Planctomycetota* MAGs encode complete 3-hydroxypropionate bicyclic or Wood-Ljungdahl pathway genes (Supplementary Fig. S9B). This suggests that the elevated dissolved inorganic carbon in the acid lake is actively incorporated into biomass by multiple phyla, potentially supporting the heterotrophic food web that sustains the unique microbial community under suboxic, acidic conditions.

Given the enrichment of halogenated compounds, we next assessed microbial pathways involved in halogen metabolism. Among the four major halogenation pathways [39], flavin-dependent tryptophan halogenase (*tdH*) showed slightly higher coverage in the acid lake whereas other halogenase classes showed no clear enrichment (Supplementary Fig. S6D, S11). Among the most abundant MAGs, S20-B6, which was predominantly found within the acid lake, did possess the gene for adenosyl-halogenase (*sadh,* Supplementary Fig. S9D), but other halogenated compound cycling genes were sparse. Because halogen metabolism remains poorly characterized, additional relevant genes may be present among currently unannotated or misannotated proteins.

Halogenated compounds may also originate from abiotic processes or organisms not captured in the sampled planktonic community. Organo-iodides are commonly produced by phytoplankton, macroalgae, and fungi through transformation of iodine salts into mainly polyketides, phenols, and alkaloids, used as antibiotics and antivirals [8]. Elevated nitrification and exopeptidases (Supplementary Fig. S11DE) suggest substantial organic-matter inputs originating from higher water layers or dissolved platforms [38], that may be metabolized releasing halogenated compounds. Haloketone dehalogenase was more abundant in the acid lake (Fig. 6E), indicating active turnover of halogenated compounds, though within the group of most abundant MAGs only S20-B6 showed the *dha* gene. However, the relatively slow metabolism of halogenated compounds combined with limited lateral mixing could provide a valid alternative hypothesis for the higher levels of halogenated compounds, given that the gene presence only provides evidence for functional potential, and transcription rates have not been measured.

Finally, we examined mechanisms of pH regulation. Na^+^/H^+^ antiporters and Na^+^/H^+^-ATPases regulate cytoplasmic pH by exporting protons [32, 51]. All *Thermoproteota* MAGs encoded Na^+^/H^+^-antiporters with U. *Nitrosopelagicus* [1] containing five copies. Within *Proteobacteria*, more abundant MAGs also contained higher numbers of these transporters. Consistently, functional profiles showed increased Na^+^/H^+^ antiporter (Fig. 6F) and Na^+^/H^+^-ATPase genes in the acid lake (Fig. 6G). A slight increase in glutamate decarboxylase was also observed (Fig. 6H), which can regulate intercellular pH by consuming protons [16].

Overall, combined metagenomic and functional analyses show that the acid lake hosts a specialized microbial community adapted to the acidity. High abundance of nitrification genes (*amo*, *hao*, *nxr*) indicates active nitrification, while halogen-metabolizing enzymes suggest ongoing turnover of naturally derived halogenated compounds. Enriched Na⁺/H⁺ antiporters, Na⁺/H⁺‑ATPases, and glutamate decarboxylase further supports effective intracellular pH regulation. Together, these traits illustrate the tightly linked microbial, chemical, and geologic processes driving the unique biochemistry of the acid lake ecosystem.

## Discussion

### Future perspectives

Exploring remote habitats has become more accessible thanks to large-scale omics datasets reducing the reliance on laboratory conditions. Yet these environments pose analytical challenges that differ markedly from those encountered in well-characterized or laboratory-controlled systems. A key difficulty lies in the holistic treatment of each sample: all detected metabolites and microbial taxa are jointly associated with the same environmental context, and consequently with one another. This obscures the directionality of causal relationships, as most conclusions are limited to co-occurrences, and our current analysis is primarily correlative. This limitation means that a mechanistic explanation is currently impossible to provide, based on the correlation network alone. Instead, the identified connections should be viewed as hypothesis-generating leads that warrant targeted validation through experiments or modeling. It is also reasonable to expect that aspects of microbial adaptation involve transcriptional changes or protein-level modifications and therefore are not captured in the functional abundance analysis. Further analysis such as protein sequence comparisons or RNA sequencing is likely to reveal additional fascinating insights within this ecosystem, if such data becomes available.

We can contemplate how our results relate to future ocean acidification. Laboratory studies report reduced nitrification rates under acidified conditions [5], yet the acid lake demonstrates that key nitrogen-cycling lineages can persist at pH levels of ~ 6 far below end-century surface ocean projections (~ 7.7–7.8). This suggests that at least some nitrifying archaea and bacteria possess sufficient plasticity to maintain function under chronic acidification. *Cyanobacteria*, which were still present at a low relative abundance within the nearly aphotic deep sinkholes (mean = 0.03, SD = 0.016), were almost completely lost in the acid lake (mean = 0.0017, SD = 0.0002), suggesting that, even when taking into account the low light conditions, photosynthetic primary producers may be more vulnerable to combined acidification and deoxygenation. Naturally, we should be careful with our interpretations, as the acid lake is nearly aphotic, higher in dissolved inorganic carbon and other nutrients, and resembles a chronically stressed, hydrodynamically isolated endpoint rather than the gradual transition expected in the open ocean, which also hopefully will never reach the extreme conditions found within the acid lake.

The Proterozoic oceans are likely closer in composition to the acid lake, as they are thought to have featured elevated CO₂, widespread suboxia, and significant microbially mediated nitrogen cycling [60, 76]. The acid lake community, with its dominance of ammonia-oxidizing archaea, suppression of oxygenic phototrophs, and active chemolithoautotrophic nitrogen cycling, may functionally resemble inferred Proterozoic communities. While not a direct reconstruction of ancient oceans, the acid lake offers a tractable modern system in which to study how microbial communities self-organize under the combined stressors that characterized Earth’s earlier ocean states.

Another consideration is the higher number of metabolites recovered from the acid lake without a definitive annotation than from other environments. Over 20% (74/327) of the LC–MS features from the acid lake could not be assigned to any structure. Until now, the metabolome of the acid lake was unexplored, which combined with databases being biased toward medical and pharmaceutical sources [47]means that homologs of the acid lake metabolites are sparse. Advances in molecular networking such as GNPS (Aron et al., 2020) and other annotation methods are currently expanding our annotation power [10]. This also emphasizes the importance of metabolomics recovered from underexplored environments. As the number and accuracy of annotations increase, the quantity and quality of connections between microbes and metabolites would also increase. Ultimately, as we continue to broaden our reference libraries and refine computational tools, the chemistry of the acid lake will likely provide a rich source of insights.

### Final remarks

This study explores a truly extraordinary marine niche, a naturally acidic, suboxic lake tucked away on the Luymes bank and reveals just how inventive its microbial residents can be. By weaving together metagenomics, untargeted metabolomics, and using analytical power of modern tools, we uncovered a microbiome dominated by nitrifying archaea and nitrite‑oxidizing bacteria, and a remarkable enrichment of organoiodides. We also identified acid‑stress defense and halogen turnover. These results not only illustrate the power of multi‑omics to decode complex, real‑world ecosystems, but also position the acidic lake as a natural laboratory for probing future ocean‑acidification scenarios and discovering novel bio‑active compounds. In short, our analysis offers a compelling glimpse into how life thrives under extreme conditions and opens exciting avenues for both fundamental research and biotechnological exploration.

## Supplementary Information

Supplementary material 1.

Supplementary material 2.

Supplementary material 3.

Supplementary material 4.

Supplementary material 5.

Supplementary material 6.

Supplementary material 7.

## Data Availability

Raw MS-data can be accessed through Massive: https://massive.ucsd.edu/ProteoSAFe/dataset.jsp?accession=MSV000101083. Metabolomics data-analysis pipeline can be found at https://github.com/MGXlab/MetabSpace. Metagenomics data can be found at European Nucleotide Archive (https://www.ebi.ac.uk/ena/browser/view/PRJEB109739). Code used for Metagenomics analysis can be found at https://github.com/MGXlab/MetagenomicAnalysisSinkhole/. All other related data can be requested from the lead contact: M.D. Boer (meineboer@gmail.com).
