# Supplementary material for: A specialized microbiome enriched in nitrification and halogen cycling potential resolved from multi-omics investigation of a Caribbean suboxic and acidic marine sinkhole
Source: Microbiome. 2026 Nov 14;14:255. doi: 10.1186/s40168-026-02564-w (PMC13769199; doi:10.1186/s40168-026-02564-w)
Supplement: Supplementary file 1 [file 40168_2026_2564_MOESM1_ESM.pdf]

Supplementary Figures

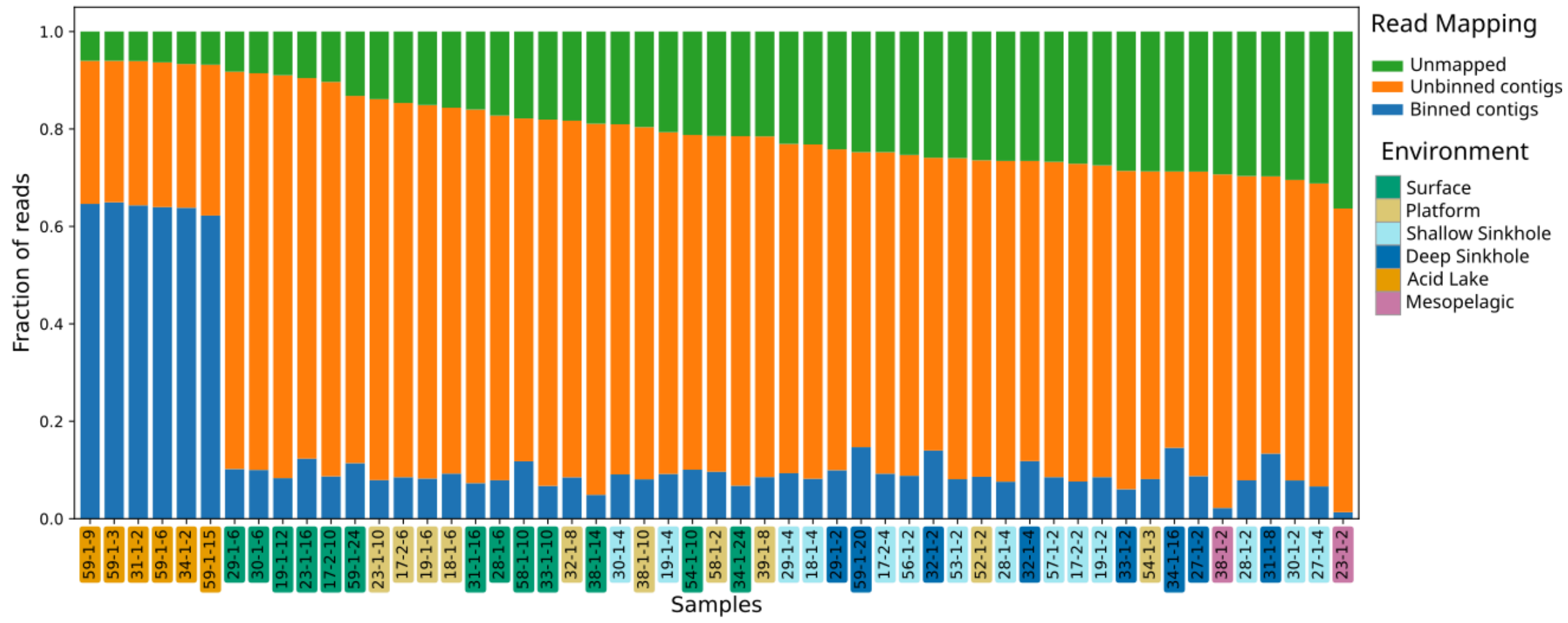

Supplementary Figure S1: Fractions of reads that could be mapped to binned (orange) and unbinned (blue) contigs. Green corresponds to the fraction of reads that were not mapped to contigs. Labels colored by environment.

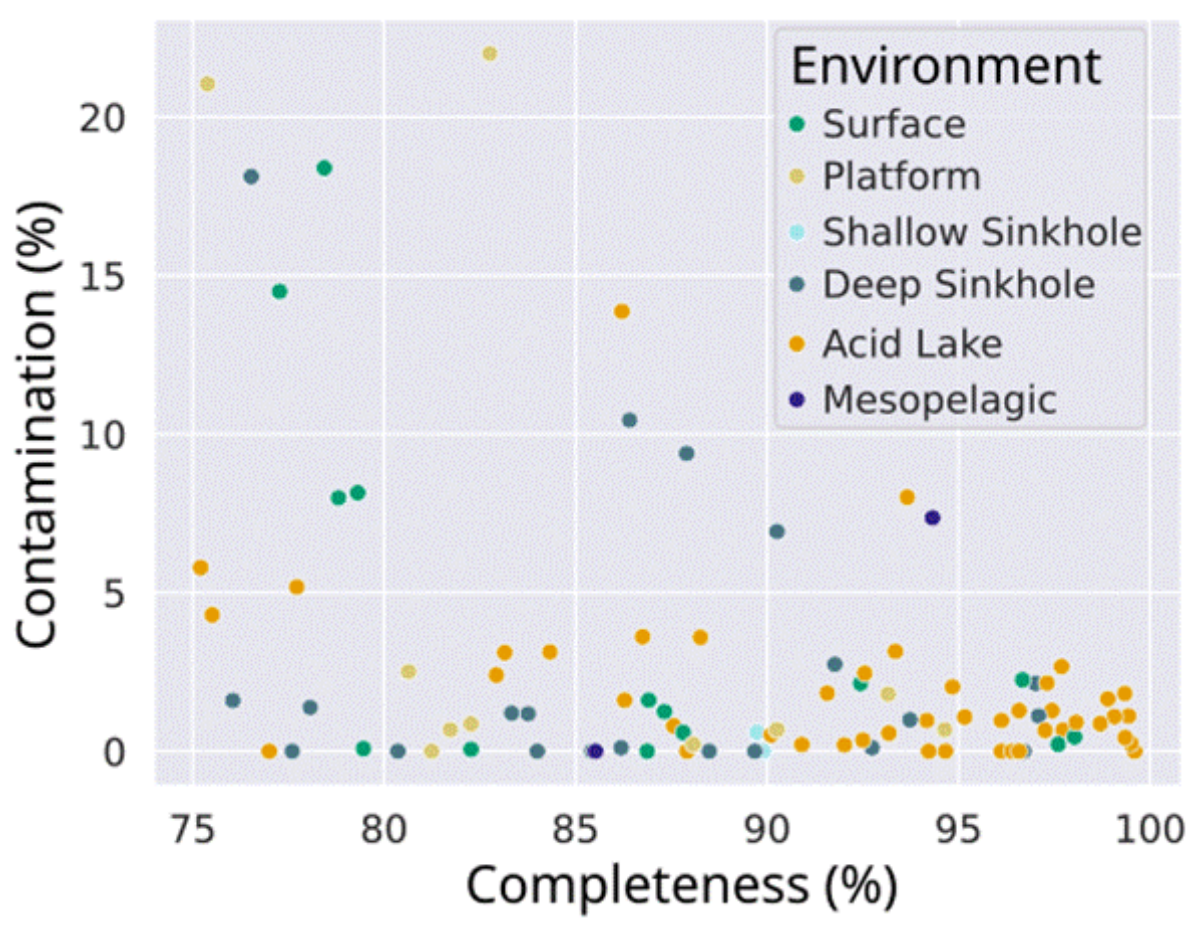

Supplementary Figure S2: Completeness-contamination plot of dereplicated MAGs colored by environment with highest mean abundance.

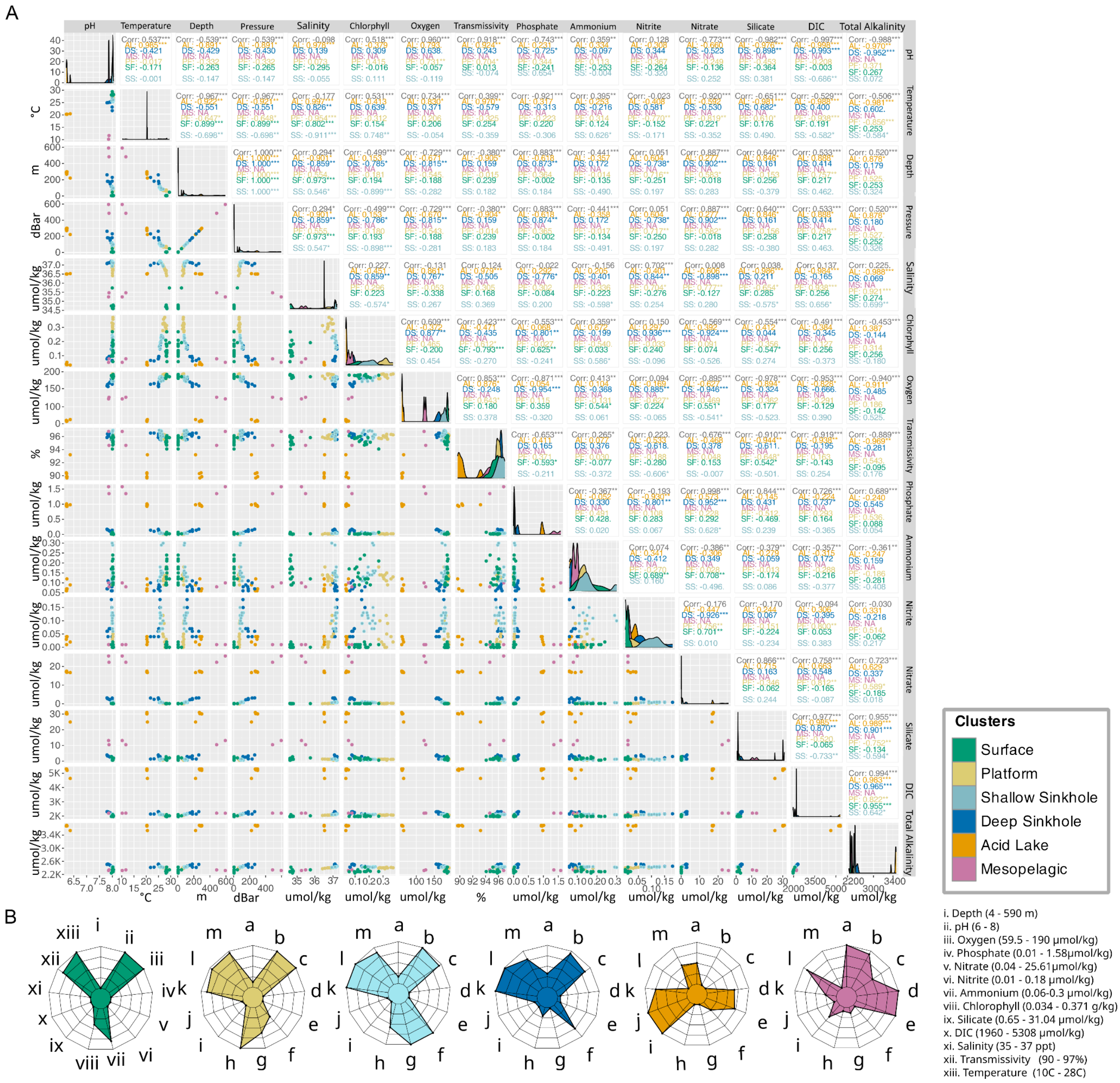

Supplementary Figure S3: Correlation plot of factors by environment. A) Top-right: total correlation and correlation within each group of abiotic factors. Bottom-left: scatterplots of factors, colored by environment. Diagonal: density distribution. B) Spider-plots of biome profiles of the six defined environments, values on the spokes were normalized to a range of 0 to 1.

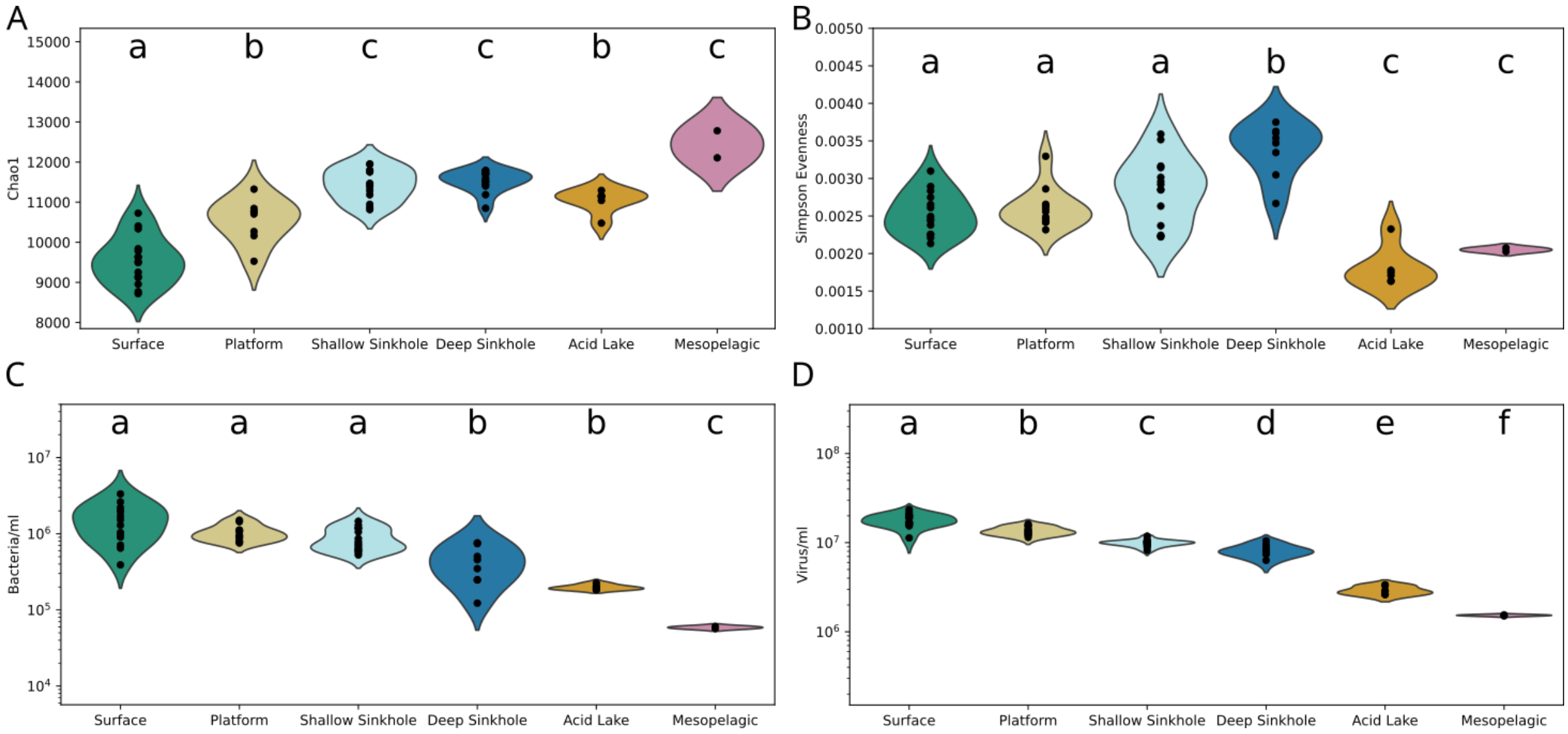

Supplementary Figure S4: Alpha diversity grouped by environments Violin plots of the A) Genus richness (Chao1) and B) Genus Evenness (Simpson) of the genera in all samples grouped and colored by environment. Violin plots of log-scale cell counts of C) bacterial and D) viral particles grouped and colored by environment. Letters correspond to significantly different groups based on Tukey-HSD test ($p < 0.05$).

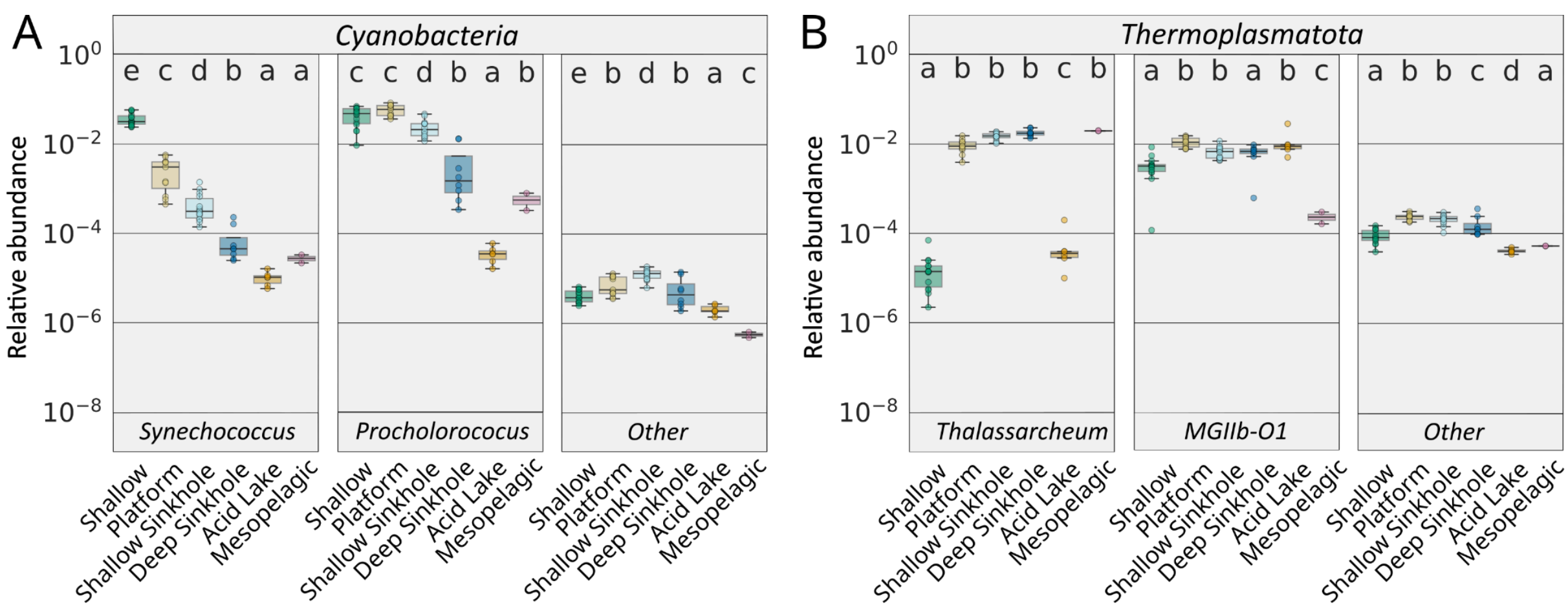

Supplementary Figure S5: Boxplots of genus abundances. Relative abundances of most abundant genera from A) Cyanobacteria and B) Thermoplasmatota. Abundances are grouped and colored by sampled environments. Letters correspond to significantly different groups (Tukey-HSD, $p < 0.05$).

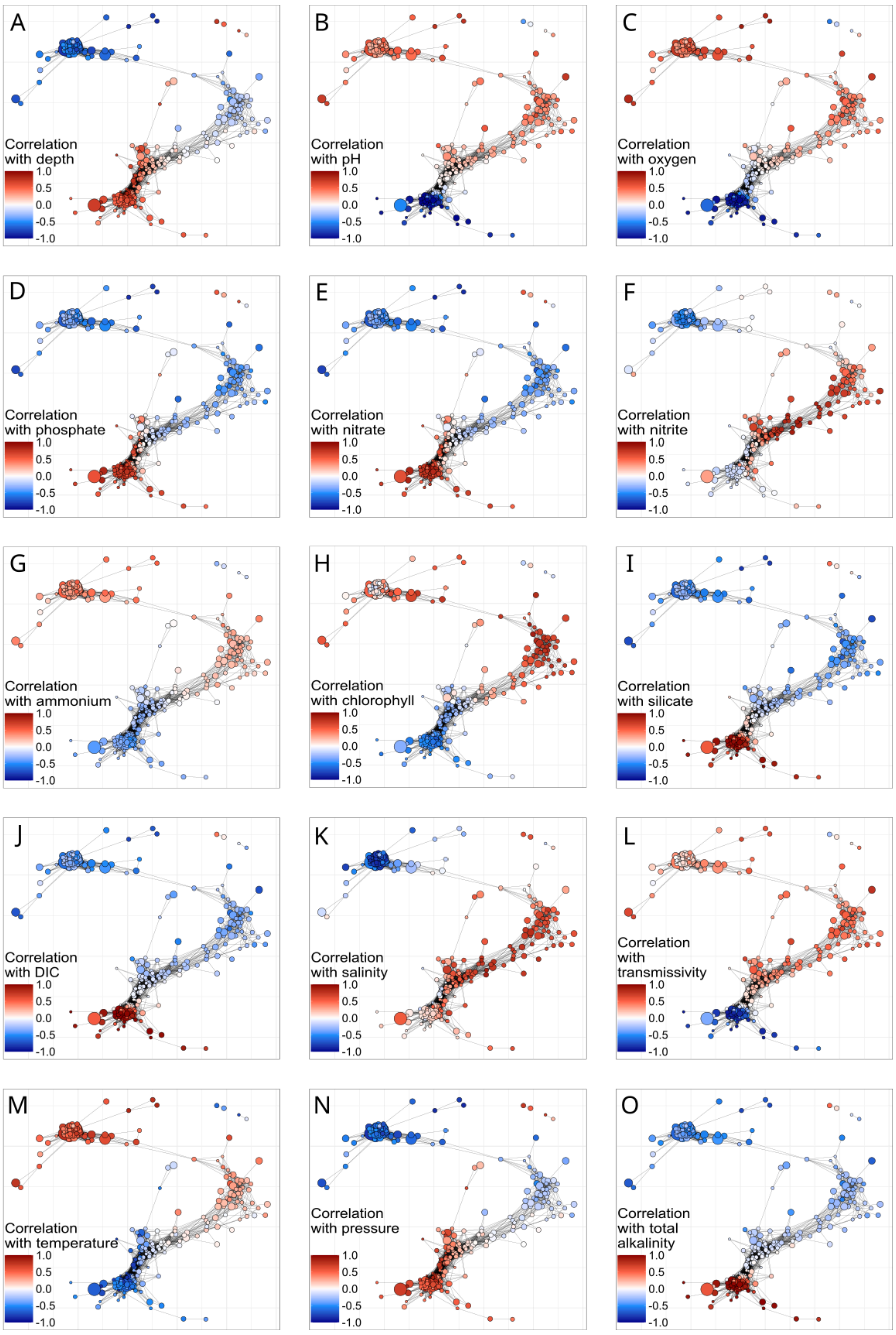

Supplementary Figure S6: SpeSpeNet plots colored by correlation with A) Depth, B) pH, C) Oxygen, D) Phosphate, E) Nitrate, F) Nitrite, G) Ammonium, H) Chlorophyll, I) Salinity, J) DIC, K) Salinity, L) Transmissivity, M) Temperature, N) Pressure, O) Total alkalinity. Networks were generated using SpeSpeNet (van Eijnatten et al., 2025). Edges show co-occurrence based on Spearman rank correlation ($\rho > 0.5$) with nodes filtered for abundance (> 0.001 in > 1 samples), prevalence and covariation (> 0 in > 4 samples), and covariation (> 1 correlation with $\rho > 0.5$). The overall layout is generated using a Fruchterman-Reingold algorithm.

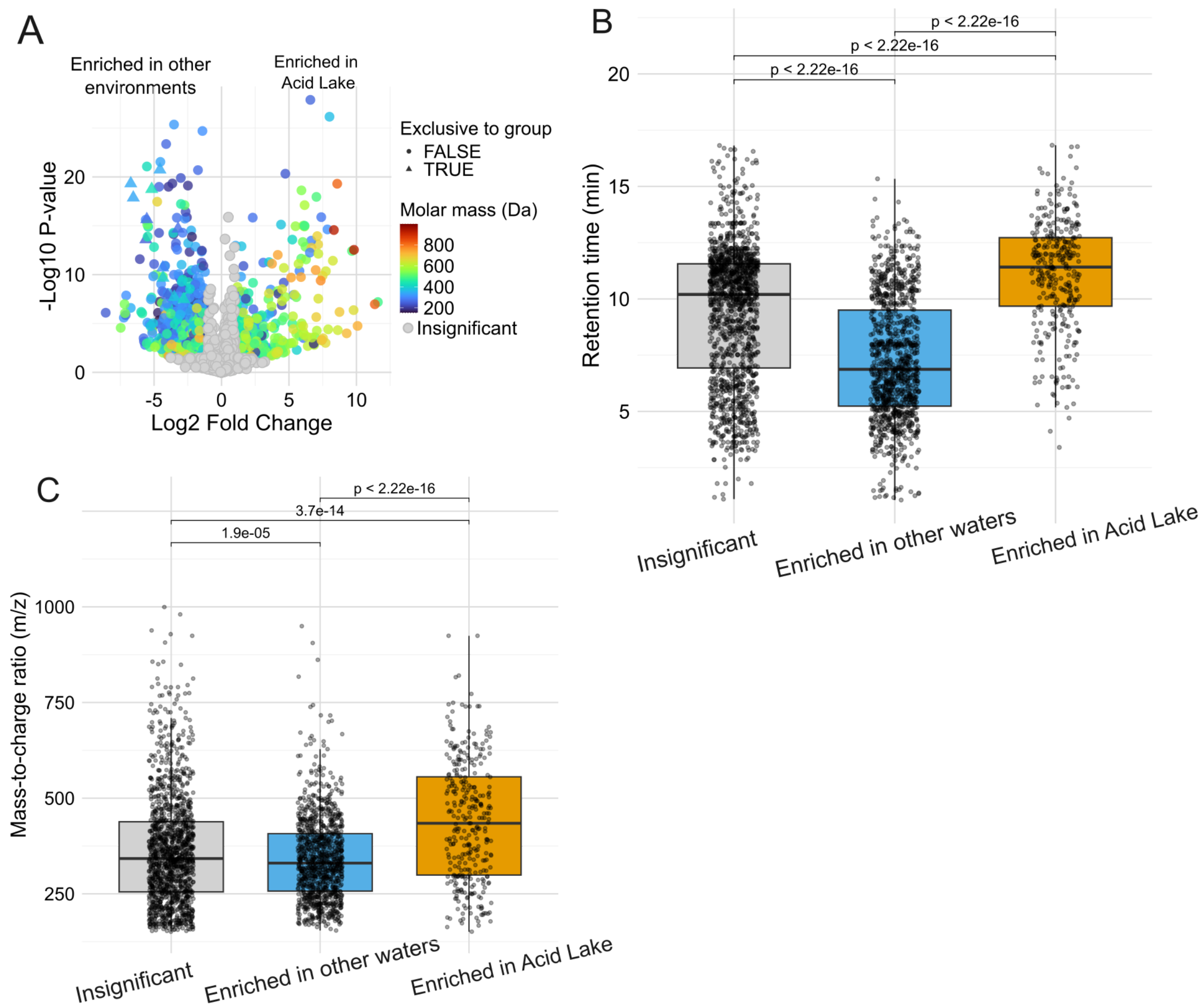

Supplementary Figure S7: A) Volcano plot of chromatographic peak areas for comparing Acid Lake to all other environments with all features colored by molar mass. Boxplots of B) Chromatographic peak retention-time and C) Chromatographic peak mass-to-charge (m/z) grouped based on enrichment in Acid Lake, all other environments and not significantly enriched (|log2-FoldChange| < 1 in either, p-values are calculated with unpaired t-test and corrected with Benjamini-Hochberg procedure (t.test() and p.adjust(p_value, method = "BH") from stats package).

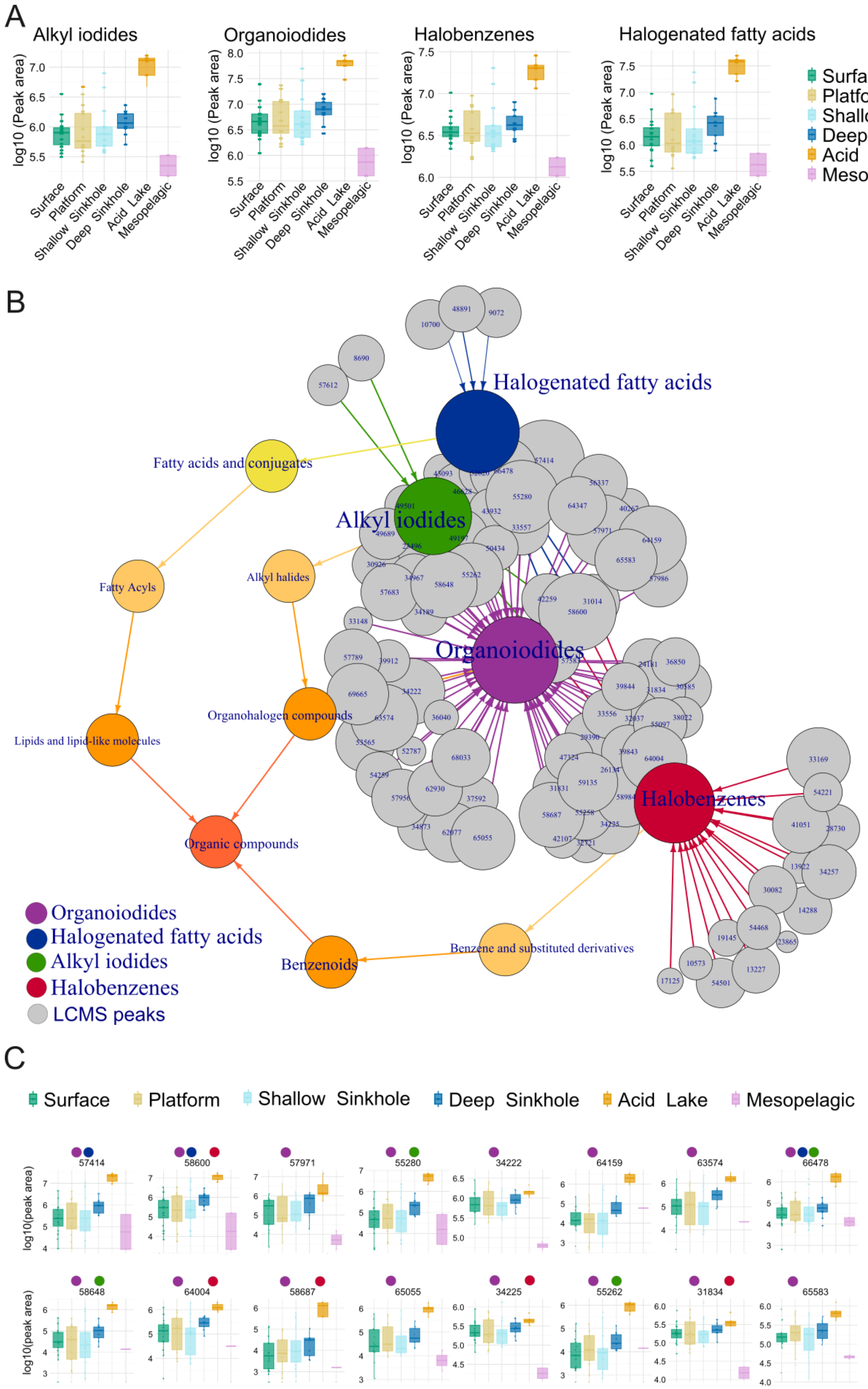
A
Alkyl iodides
Organoiodides
Halobenzenes
Halogenated fatty acids
log10 (Peak area)
Surface
Platform
Shallow Sinkhole
Deep Sinkhole
Acid Lake
Mesopelagic
B
Halogenated fatty acids
Fatty acids and conjugates
Alkyl iodides
Fatty Acyls
Alkyl halides
Organoiodides
Lipids and lipid-like molecules
Organohalogen compounds
Halobenzenes
Organic compounds
Benzene and substituted derivatives
Benzenoids
Organoiodides
Halogenated fatty acids
Alkyl iodides
Halobenzenes
LCMS peaks
C
Surface
Platform
Shallow Sinkhole
Deep Sinkhole
Acid Lake
Mesopelagic
log10(peak area)
57414
58600
57971
55280
34222
64159
63574
66478
58648
64004
58687
65055
34225
55262
31834
65583

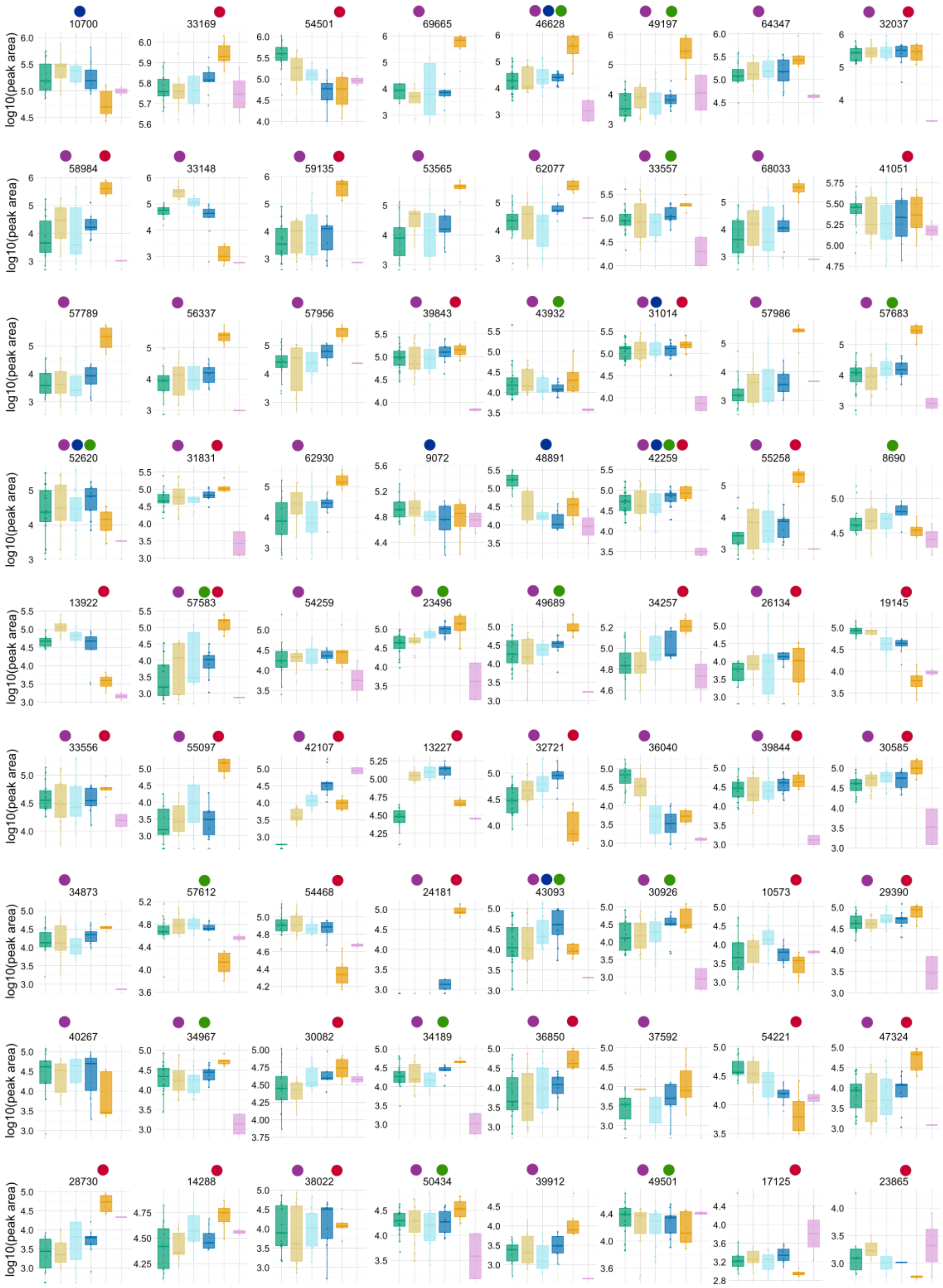

Supplementary Figure S8: A) Summed LC-MS/MS ion log10 peak areas of four halogenated CANOPUS classes which showed enrichment in Acid Lake. B) Network of the classification of the four enriched CANOPUS classes, showing hierarchical structure and shared categorization of features. Node size corresponds to log10 peak area. C) Log10 peak areas of all features that are a member of at least one of the four halogenated compounds classes. Colored dots show membership of the different CANOPUS classes.

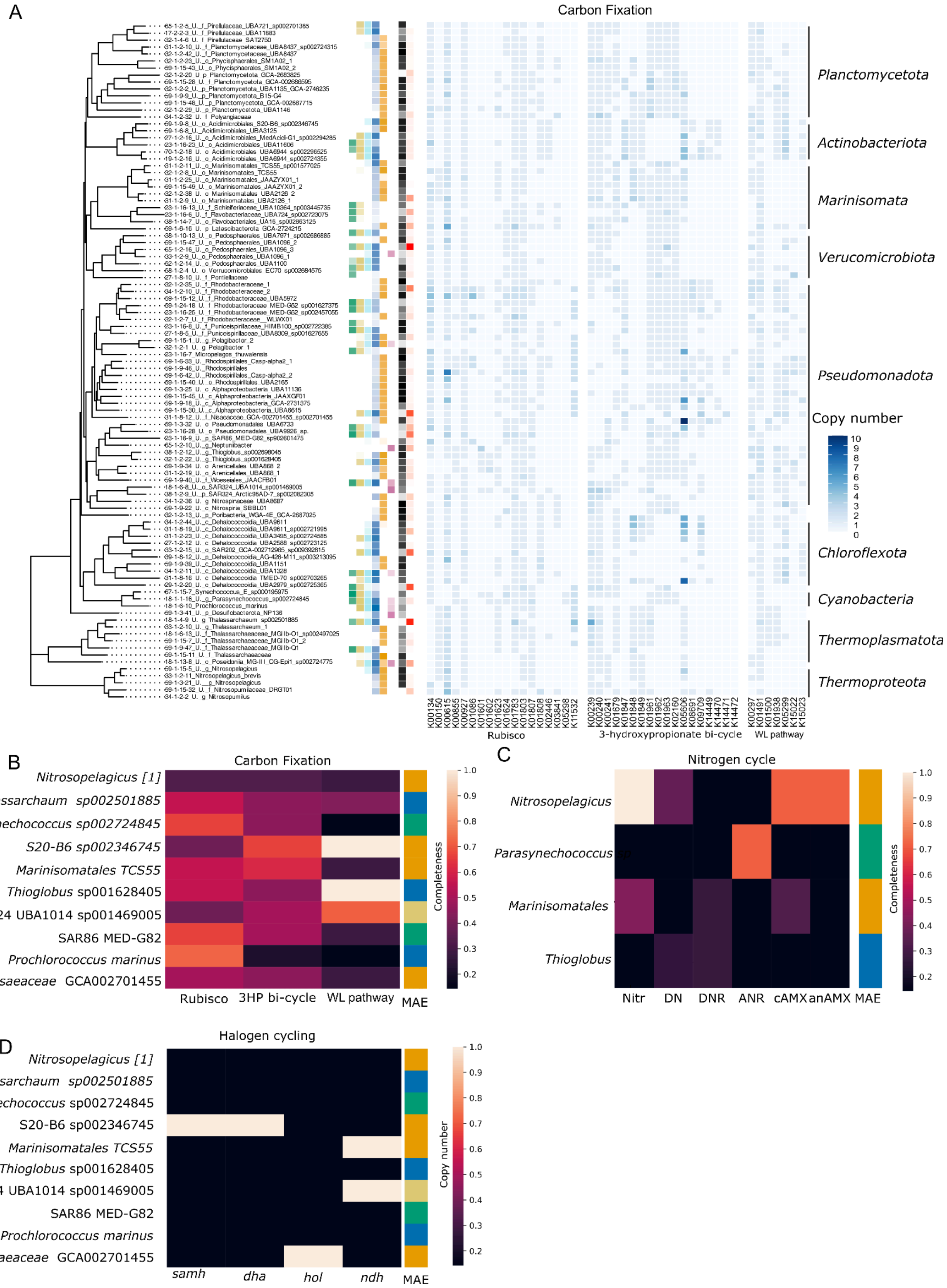

Supplementary Figure S9: A) Phylogenetic tree of 102 dereplicated MAGs based on concatenated alignment of 396 marker genes using Phylophlan (Asnicar et al., 2020) with default parameters. B) Carbon pathway completeness and C) Nitrogen cycle completeness and D) Halogen cycling genes within the top 10 most abundant MAGs. Heatmaps show KO gene counts (top) and carbon/nitrogen pathway completeness scores (bottom) across

diverse metagenome-assembled genomes. MAE = Most Abundant Environment based on highest MAG abundance.

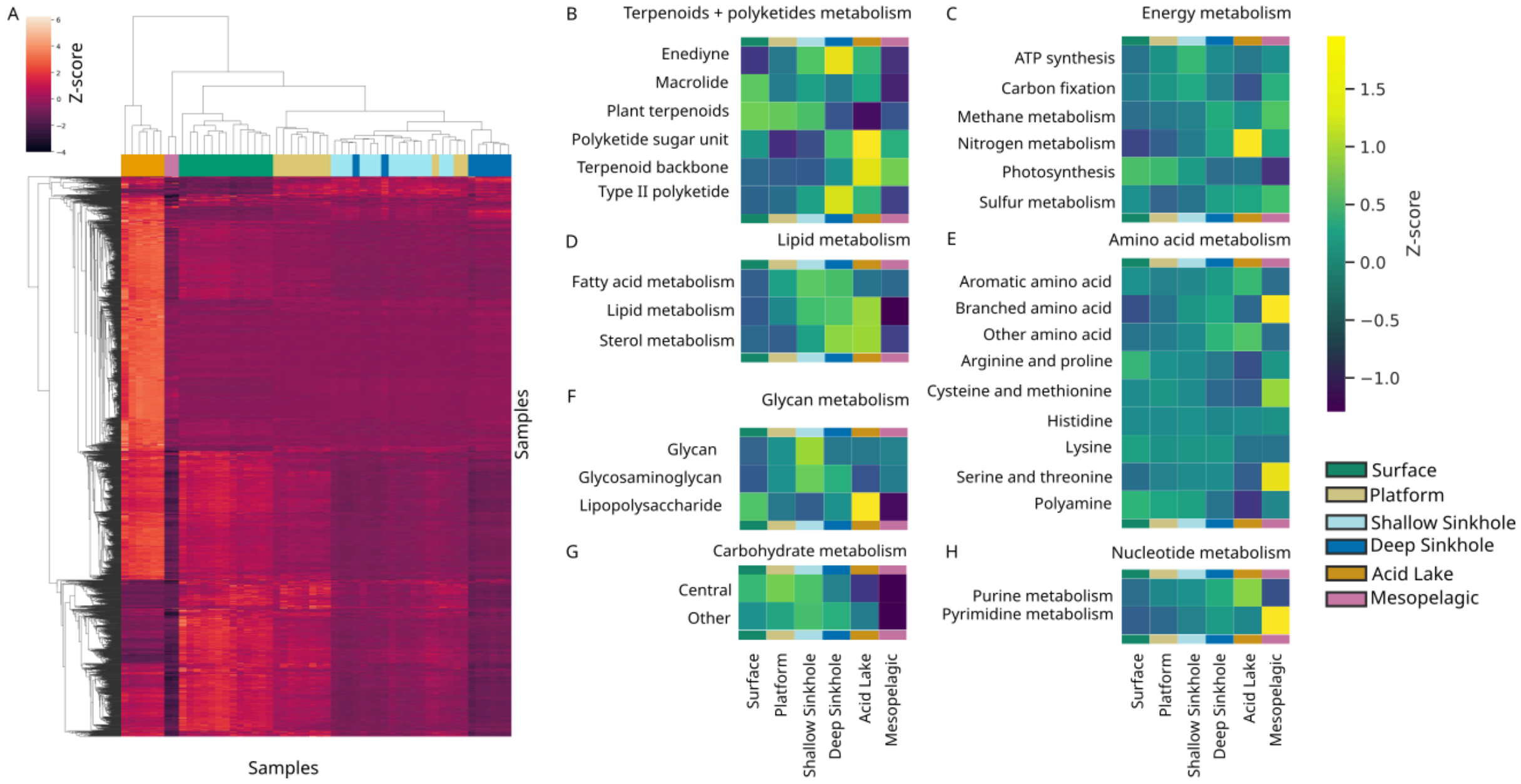

Supplementary Figure S10: A) Z-score normalized abundances of KEGG orthologs (KOs), columns are samples labeled by environment with color. B-F Z-score normalized abundances of KEGG modules: B) Terpenoids and Polyketides Metabolism, C) Energy metabolism, Lipid metabolism, E) Amino acid metabolism, F Glycan metabolism, G) Carbohydrate metabolism and H) Nucleotide metabolism.

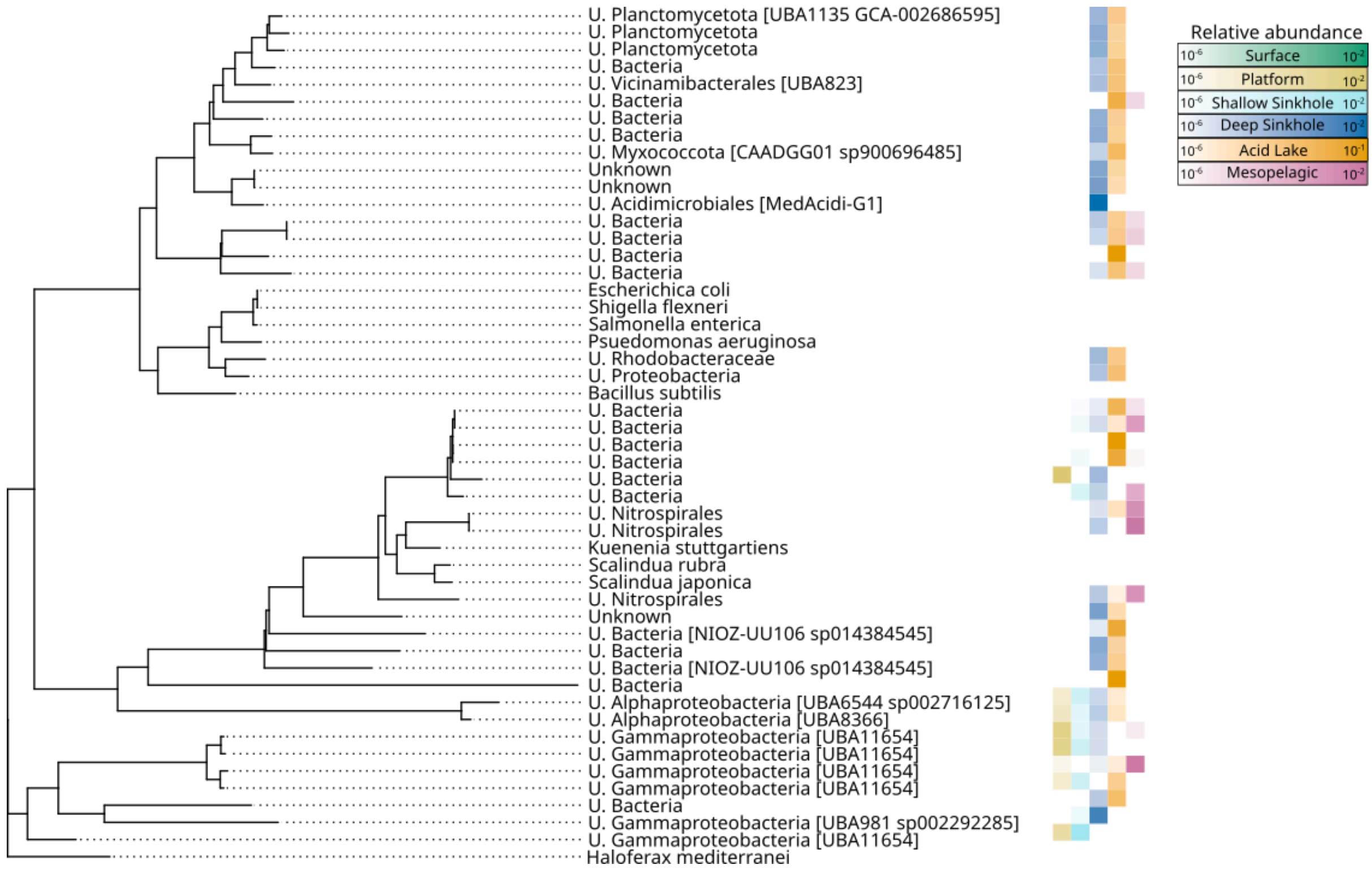

Supplementary Figure S11: nar/nxr gene tree based on multiple sequence alignment of the protein sequences of the representatives from all protein sequences annotated with nar/nxr. Taxonomic annotation of the sequence tree is based on the cluster representative. Heatmap shows normalized abundance calculated based on the summed coverage of all cluster members averaged over the different environments. Branches without heatmap are reference sequences.

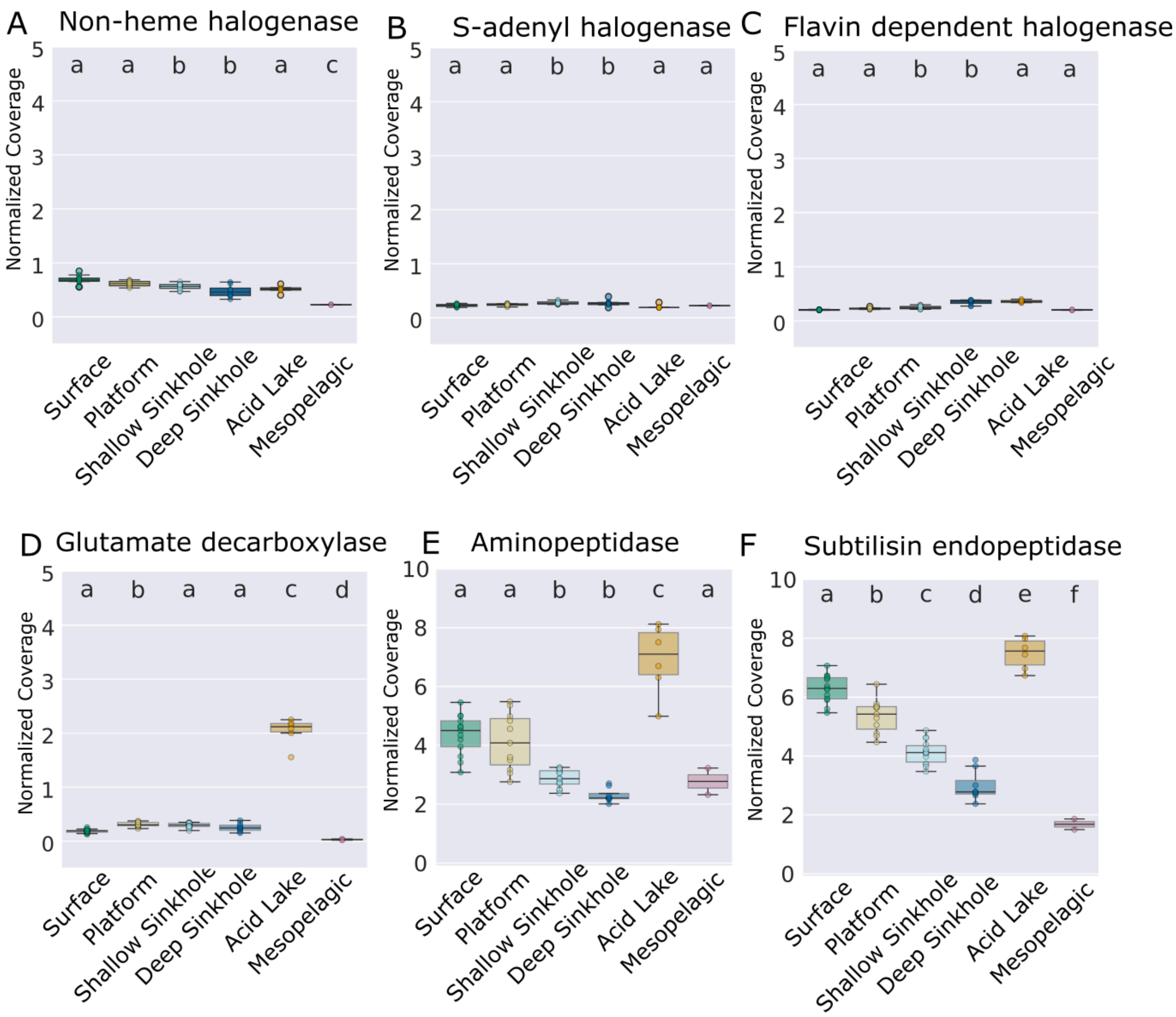

Supplementary Figure S12: Functional annotation of contigs continued. Normalized coverage of A) Glutamate decarboxylase B) Tryptophan halogenase. C) Aminopeptidase D) Endopeptidase. Normalized coverage is calculated based on RAT, summed by sample, averaged over the environment. Letters show significantly different environments (TukeyHSD: $p < 0.05$).

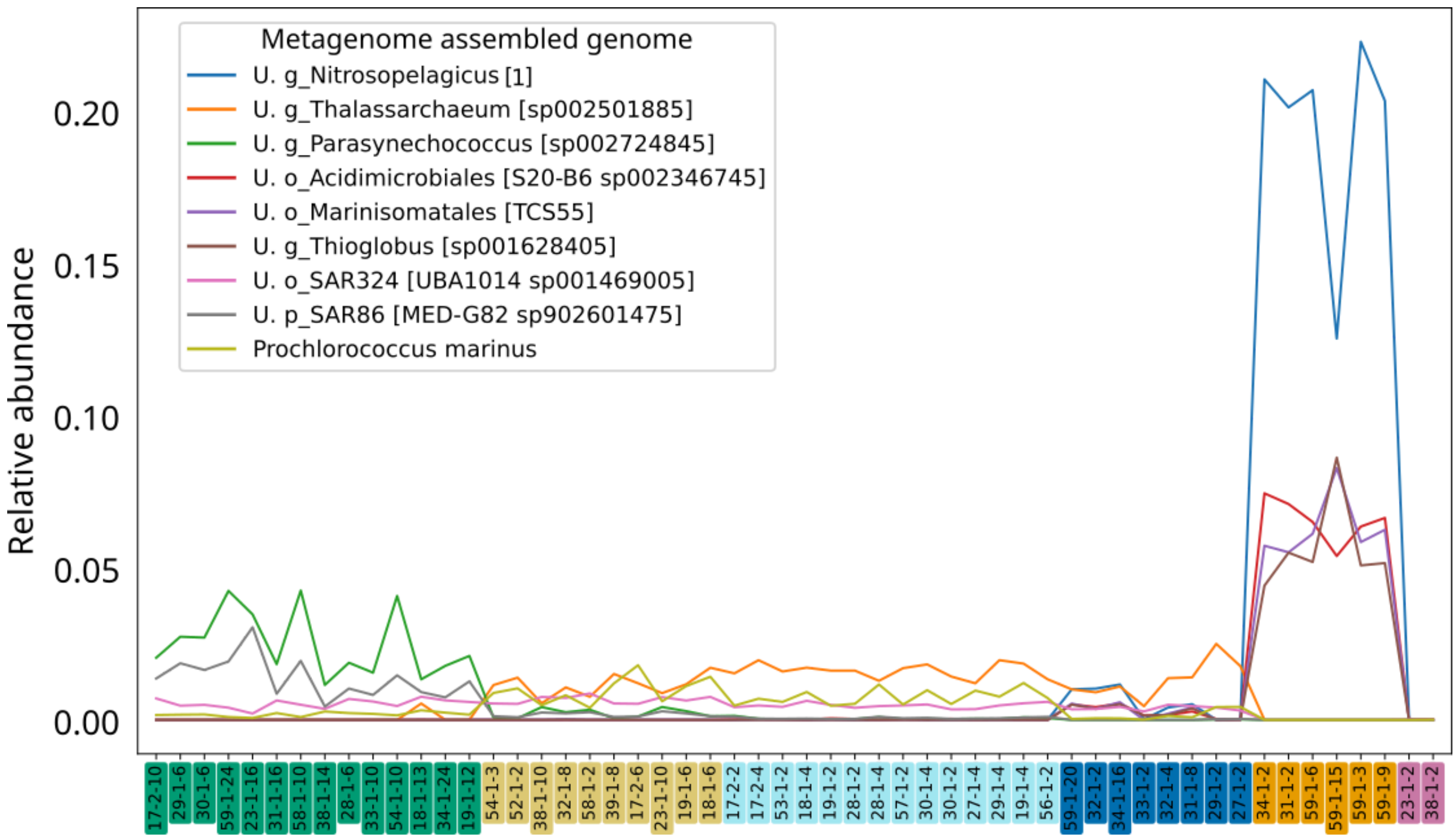

Supplementary Figure S13: abundances top10 MAGs: Relative abundance of the 10 most abundant MAGs in all samples. Labels colored and ordered by environment.

Supplementary Tables

Supplementary Table S1: Statistics and quality metrics metagenomes. [Excel Table]

Supplementary Table S2: Dereplication clustering details, clusters, cluster representatives, completeness, contaminated. Dereplicated Metagenome-Assembled Genomes (MAGs). [Excel Table]

Supplementary Table S3: Physiochemical measurements. [Excel Table]

Supplementary Table S4: Selected important compound classes enriched in Acid Lake. Selection done with Recursive Feature Elimination (RFE).

Canopus absoluteIndex SIRIUS	Name	Amount of LC-Ms features per class
Canop1356	Acyclic diterpenoids	5 features
Canop35	Halobenzenes	41 features
Canop64	Macrolactams	11 features
Canop1028	Alkyl iodides	22 features
Canop1517	Organoiodides	69 features
Canop3537	Methyl-branched fatty acids	69 features
Canop3082	Medium-chain fatty acids	74 features
Canop348	Peptides	25 features
Canop4157	Phenolic glycosides	2 features
Canop338	Branched fatty acids	178 features
Canop4097	1,3,5-triazines	8 features
Canop261	Phenylpropanoids and polyketides	43 features
Canop111	Tetrazoles	10 features
Canop3092	Halobenzoic acids and derivatives	1 feature
Canop281	Aminobenzoic acids and derivatives	1 feature

Canop483	Halogenated fatty acids	11 features
Canop2717	Lupin alkaloids	1 feature
Canop4801	Alpha-chloroketones	1 feature
Canop4800	Alpha-haloketones	1 feature
Canop3096	4-halobenzoic acids and derivatives	1 feature
Canop3094	2-halobenzoic acids and derivatives	1 feature
Canop226	Thiazolidines	1 feature
Canop3881	Vinylogous halides	1 feature

Supplementary Table S5: Canopus classes and taxonomy [Excel Table]

Supplementary Table S6: Functional annotation of MAGs and contigs [Excel Table]

Supplementary Table S7: Taxonomics-metabolomics correlations [Excel Table]

Supplementary Table S8. Used MZmine parameters.

MZmine 4.2.3	
Crop filter	50-1500 Da
Mass detection	MS1 - 5E4; MS2 - 5E3
ADAP Chromatogram Builder	Min cons scans = 5, ADAP intensity both 5E4. Tolerance 0.002 or 10 ppm
Local minimum feature resolver	MS/MS pairing on, threshold 90%, min search 0.04, height 0%, min absolute height 5E4, ratio peak 1.70, peak duration 0-10, min scan point 5
13C isotope filter	0.001 or 5ppm, retention time tolerance 0.1 min, monotonic shape off, max charge 2
Alignment -> Join Aligner	m/z: 0.002 or 10ppm, weight 3, RT - 0.4min, weight 1
Feature list blank subtraction	Min detection in blank = 2
Gap filling -> Feature Finder(multithread)	20%, 0.001 or 5ppm, 0.4 min, minimum 3 scans
Duplicate peak filter	m/z 0.001 or 2 ppm, 0.2min tolerance

Supplementary S9. Used SIRIUS parameters

config --IsotopeSettings.filter=true --FormulaSearchDB= --Timeout.secondsPerTree=0 --FormulaSettings.enforced=HCNOFSiPI --Timeout.secondsPerInstance=0 --AdductSettings.detectable=[[M-H2O+H]+,[M+K]+,[M-H4O2+H]+,[M+H]+,[M+H3N+H]+,[M+Na]+] --UseHeuristic.mzToUseHeuristicOnly=650 --AlgorithmProfile=orbitrap --IsotopeMs2Settings=IGNORE --MS2MassDeviation.allowedMassDeviation=10.0ppm --NumberOfCandidatesPerIon=1 --UseHeuristic.mzToUseHeuristic=300 --FormulaSettings.detectable=B,Cl,Br,Se,S --NumberOfCandidates=5 --AdductSettings.enforced=, --AdductSettings.fallback=[[M+K]+,[M+H]+,[M+Na]+] --FormulaResultThreshold=true --InjectElGordoCompounds=true --StructureSearchDB=BIO,METACYC,CHEBI,COCONUT,ECOCYCMINE,GNPS,HMDB,HSDB,KEGG,KEGGMINE,KNAPSACK,MACONDA,MESH,NORMAN,UNDP,PLANTCYC,PUBCHEM,PUBMED,YMDB,YMDBMINE,ZINCBIO --RecomputeResults=false formula fingerprint structure canopus

Supplementary methods

Nutrient and physiochemical measurements

Depth, salinity, temperature, density, and O2 concentrations were measured using a CTD rosette equipped with a Nortek Aquadopp Profiler and a O2-optode. Measurements for phosphate, ammonium, nitrate with nitrite (combined) and nitrite were taken on board using a Seal QuAAtro, a gas-segmented continuous flow auto analyzer. At selected sites, Dissolved Inorganic Carbon (DIC) samples were collected following Dickson et al. (2007) and both DIC and Total Alkalinity (TA) were analyzed on a VINDTA 3C calibrated using certified reference material batch 171 obtained from Prof A. Dickson (Scripps Institution of Oceanography, USA). Additionally, water (2000 ml) was collected immediately from the water sampling bottles after each rosette deployment and prepared for simultaneous measurements of microbial community, Dissolved Organic Carbon (DOC), and solid phase extraction of Dissolved Organic Matter (DOM) as described in Sparagon et al. (2024). A peristaltic pump connected to acid-washed and seawater-leached silicon tubing was used to pass 1200 ml of sampling water through a 0.22 µm Sterivex to collect bacterioplankton for downstream DNA analysis. For the analysis of metabolites, 1000 mL of the remaining 0.22 µm Sterivex filtrate was collected in acid-washed polycarbonate bottles and acidified with HCl to pH < 2. A small volume (50 mL) of the acidified sample water was used to flush the lines prior to the solid phase extraction, resulting in 1000 mL of sample for solid phase extraction following the protocol described by Petras et al. (2017). Sterivex filters were immediately frozen (-80 °C) for later DNA extraction.

Nutrient measurements were taken on board using a Seal QuAAtro, a gas-segmented continuous flow autoanalyzer operated in an air-conditioned lab container. Measurements were made simultaneously for phosphate, ammonium, nitrate with nitrite (combined) and nitrite only. All measurements were calibrated with stock standards diluted in low-nutrient seawater in the same salinity range as the samples. At some stations, sub-samples were collected and preserved for Dissolved Inorganic Carbon (DIC). These samples were collected following an established protocol (Dickson, A. G., Sabine, C. L., and Christian, 2007). DIC and total alkalinity (TA) using two separate instruments: a VINDTA 3C (for both DIC and TA) and an in-house spectrophotometric titration system (for TA only). The DIC and TA results, again, were calibrated using regular measurements of batch 171 certified reference material (CRM) seawater obtained from the laboratory of Prof. A. G. Dickson.

The measurement of these components of the marine carbonate system allows us to calculate seawater properties, including pH. This was calculated using a macros Excel file

with a built-in formula. This program takes two parameters of the CO2 system in seawater (in this case TA and DIC), factors in the local conditions (input conditions), and calculates the pH along with a set of other non-relevant outputs. The required input includes the local salinity, temperature and pressure, the concentrations of silicate and phosphate, and the two known CO2 system parameters. TA and DIC, which do not vary with temperature and pressure, are used to calculate the pH.

DNA isolation

DNA was extracted with a NucleoSpin Tissue DNA extraction kit following manufacturer instructions with the following adjustments. Each filter was thawed at room temperature, air-dried with a Luer lock syringe, and closed on one end. Subsequently, 410 µL of proteinase K (50 µL) and buffer T1 (360 µL) solution was pipetted into the Luer lock opening of the filter, which was then closed and rotated overnight (~18 hours) at 55°C in a hybridization oven (Compact Line OV4, Biometra). On the next day, 400 µL of Buffer B3 was added to the filters, and filters were rotated at 70°C for 15 min. Subsequently, the liquid (500–750 µL) was removed from the filters with 3 mL Luer lock syringes and placed into a microtube. Samples were diluted with pure ethanol in a volume ratio of 1:2 ethanol:sample and vortexed. The solution was then added to the column and centrifuged for 1 min at 11,000 × g. After washing the column with 500 µL of BW and 600 µL of B5 buffers, the silica membrane containing the extracted DNA was dried for 2 min at 11,000 × g. Pure DNA was then eluted into a microtube with 100 µL BE buffer which was incubated for 10 min at room temperature and spun down for 1 min at 11,000 × g. The concentration of isolated dna was 1.7-12 ng/µL and subsequently sequenced on the Illumina NovaSeq 6000 platform.
